# Advances on anticancer fungal metabolites: sources, chemical and biological activities in the last decade (2012–2023)

**DOI:** 10.1007/s13659-024-00452-0

**Published:** 2024-05-14

**Authors:** Antonio Evidente

**Affiliations:** Institute Biomolecular Chemistry, National Research Council, Via Campi Flegrei 34, 80078 70125 Pozzuoli, NA Italy

**Keywords:** Fungi, Metabolites, Chemical characterization, Anticancer activity

## Abstract

Among microorganisms, fungi are the ones that have the most imagination in producing secondary metabolites with the most varied structural differences, which are produced through different biosynthetic pathways. Therefore, they synthesize secondary metabolites classifiable into numerous families of natural compounds such as amino acids, alkaloids, anthraquinones, aromatic compounds, cyclohexene epoxides, furanones, macrolides, naphthoquinones, polyketides, pyrones, terpenes, etc. They also produced metabolites with very complex structures that can not be classified in the known families of natural compounds. Many fungal metabolites show different biological activities with potential applications in agriculture, food chemistry, cosmetics, pharmacology and medicine. This review is focused on the fungal secondary metabolites with anticancer activity isolated in the last ten years. For some metabolites, when described, their biosynthetic origin, the mode of action and the results of structure activity relationships studies are also reported.

**Graphical Abstract:**

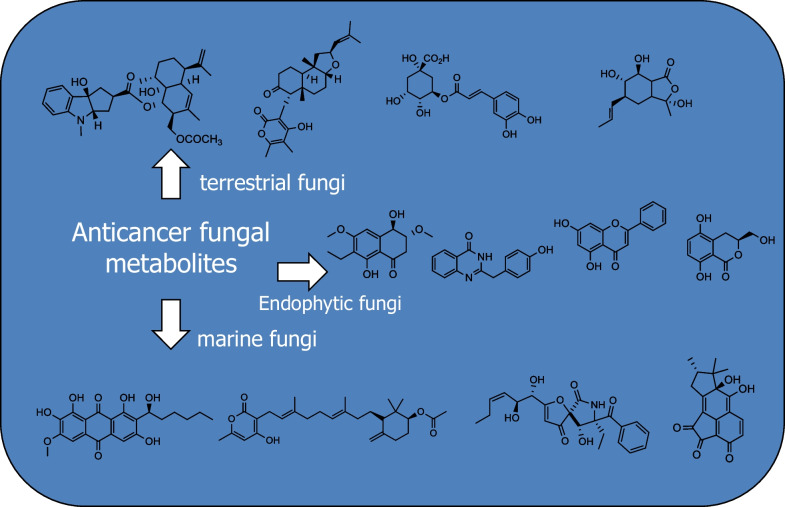

## Introduction

Bacteria and plants metabolites have played an important role in the discovery of new anticancer drugs considering the large number of derived drugs clinically used. Among these anticancer substance there are doxorubicin, daunomycin, mitomycin C, bleomycin, all synthesized by *Streptomyces*, or etoposide, teniposide, topotecan, paclitaxel and the vinca alkaloids, as vincristine, vinorelbine, extracted from plant. Also fungi are an important source to obtain new drugs as antibacterial penicillins, cholesterol-lowering lovastatin, antifungal echinocandin B, and immunosuppressive cyclosporin A, which are all drugs of fungal origin.This is in agreement with data reported in the last review of Newman and Cragg [1] who also considering, mimic, natural derivatives, synthetic inspired and synthetic containing natural chromophore compounds. Surprisingly, although a large number of fungi-derived compounds with promising anticancer activity were discovered, as well as their synthetic derivatives, a reduced number of fungal metabolites was admitted as a clinical cancer drug. However, metabolites isolated from medicinal fungi may be considered an important group of new anticancer agents [2, 3] and many patented anticancer compounds have been obtained from terrestrial fungi [4].

 This kind of metabolites, regulate a large set of biological processes including apoptosis, angiogenesis, metastasis, cell cycle regulation, and signal transduction cascades [5]. In the past but also in the last decade (2012–2023), which is an object of the present work, many reviews have treated the fungal compounds with potential anticancer activity but only partially dealing the argument. Earlier one of them discussesed the potential anticancer activity but only of phytotoxins isolated from phytopathogenic fungi [6]. Successively, another one describes the substances of the secondary metabolism of *Epicoccum* spp., and their biotechnological potential. Among the bioactive compounds produced by this fungal genus, there are epicocconone, which is commercially available as a telomerase inhibitor, and taxol, which is a drug to treat cancer, originally isolated from *Taxus brevifolia* [7]. Among the several reviews published in this decade, just in the last year, a review reports on the anticancer compounds produced by endophytic fungi and bacteria [8], while another one is a miscellaneous description of metabolites with potential anticancer activity isolated from cultivated plants and marine bacteria and fungi [9]. Regarding the most significant past reviewes treating the fungal metabolites with anticancer activity it need to cited that published by Kim and Dewapriya [10] which include metabolites from the fungal with different origin and some already used in clinical phases I and II studies [11] and that discussing about the metabolites produced by endophytic fungi [12].

In the time fungi have demonstrated to be an excellent source of bioactive compounds with increasing potential application in different field as agriculture [13] synthesing thousand and thousand metabolites with original carbon skeletons and also differently functionalized. They include aromatic compounds, amino acids, anthracenones, butanolides, butenolides, cytochalasans, macrolides, naphthalenones, pyrones, terpenes, etc. [14–17]. These original properties could allow that new natural compounds overcome the resistance developed by plant, micorganisms, virus etc. which is became an alarming epidemiological emergence of WHO (World Health Organization). They predict that if no action is taken, after 2050, deaths from previously treatable infections will be 10 million per year [18]. A limitation in the development of formulates based on fungal bioactive metabolites and their practical application, is not due to their lower yield production but to the lacking of easy biotechnological methods to yield them in large scale, in respect to those used to produce bacterial and plant derived products. However, many efforts are done to overcome this important limiting feature [19].

The present review reports the source, the chemical characterization and the anticancer activity of metabolites isolated in the last decade (2012–2023) from terrestrial, endophytic and marine fungi. When described also the partial or total synthesis of some metabolites will be discussed as well as their mode of action. In each section the metabolites were chronologically discussed except those treating close arguments.

## Metabolites from terrestrial fungi

Fungi can be devide in toxigenic and endophytic microrganisms (Tables 1 and 2). Those belonging to the first group produce toxins responsible of severe diseases against humans, plants, other microorganisms, insects etc. The endophytic fungi do not synthesize toxins. However, the present section describes essentially the metabolites with anticancer activity produced by terrestrial toxigenic fungi and in particular those synthesized by microorganisms pathogen for plants. An interesting review on this argument was published by Evidente et al. [6]. This section will not treat of the anticancer activity of fusicoccin, the main phytotoxic metabolite produced by *Phomopsis amygdali*, the agent responsible of peach and almond disease, as its chemical and the biological aspects were recently reviewed [20]. The same is for ophiobolin A, a sesterterpene which share with fusicoccin the typical 5:8:5 carbotriciclic ring system, and sphaeropsidin A, a pimarane diterpene. These two phytotoxins, which are produced by several fungi belonging to *Bipolaris* and *Diplodia* genera, respectively, which have been estensively studied for their chemical and biological activity including the anticancer one and for the mode of action, have been object of a review recently published on Nat. Prod. Rep. [21].

**Table 1:** Metabolites (**1**–**28**) from terrestrial fungi

Compound	Fungal producer	Other biological activities	Refs.
Fischerindoline (**1)**	*Neosartoria pseudofischeri*	Not reported	[22]
Eurochevalierine (**2**)	“	“	[22, 24, 25]
Pyripyropene E (**3**)	“	“	[22]
Gliotoxin (**4**)	“	“	“
Higginsianin A (**5**)	*Colletotrichum higginsianum*	“	[26, 28]
Higginsianin B (**6**)	“	“	“
Higginsianin D (**7**)	“	“	[29]
Higginsianin E (**8**)	“	“	“
Chlorogenic acid (**9**)	*Screlotium rolfsii*	Antioxidant, antimicrobial, anti-inflammatory and hepatoprotective	[32]
Radicinin (**10**)	*Cochlobolus australiensis*	Phytotoxic	[35]
Radicinol (**11**)	“	“	“
3-*epi*-Radicinol (**12**)	“	“	“
Cochliotoxin (**13**)	“	“	“
Massarilactone D (**27**),	*Kalmusia variispora*	“	[39]
Massarilactone H (**28**),	“	“	“

“: identical data to the entry immediately above

**Table 2:** Metabolites (**29**–**66**) from endophytic fungi

Endophytic terrestrial fungi			
Compound	Fungal producer	Other biological activities	Refs.
Pestheic acid (**29**)	*Pestalotiopsis guepinii*	Plant growth regulator Not reported	[43] [45]
Botryoisocoumarin A (**30**)	*Botryosphaeria* sp.	“	[46]
Helicascolide F (**31**)	*Talaromyces assiutensis* JTY2	Antifungal	[48]
Talaromydine (**32**)	“	“	“
Episorin A (**33**),	*Epicoccum sorghinum*	Antifeedant, antifungal, cytotoxicity	[49]
Epicosorin A (**34**)	“	Anitfeedant, inhibition of NO production, anti-acetylcholinesterase	“
Epicosorin B (**35**)	“	“	“
Epicosorin C (**36**)	“	Antifeedant	“
Epicosorin D (**37**)	“	“	“
Epicohin (**38**)	“	“	“

Endophytic marine fungi			
Compound	Fungal producer	Other biological actvities	Refs.
8-Hydroxy-2-[1-hydroxyethyl]-5,7-dimethoxynaphtho[2,3-b] thiophene-4,9-dione, (**39**)	*Aspergillus terreus*	Not Reported	[50]
Anhydrojavanicin (**40**)	“	Inhibition of AChE	“
8-*O*-Methylbostrycoidin (**41**),	“	“	“
8-O-Methyljavanicin (**42**)	“	Nor reported	“
Botryosphaerone D (**43**)	“	“	
6-Ethyl-5-hydroxy-3,7-dimethoxynaphthoquinone (**44**)	“	Not reported	“
3β,5α-Dihydroxy-(22*E*,24*R*)-ergosta-7,22-dien-6-one (**45**)	“	“	“
3β,5α,14α-Trihydroxy-(22*E*,24*R*)-ergosta-7, 22-dien-6-one (**46**),	“	“	“
NGA0187 (**47**)	“	Inhibition of AChE Neurite outgrowth in PCl2 cells	[50] [51]
Beauvericin (**48**)	“	Inhibition of AChE	[50]
Chrysotriazole A (**49**)	*Penicillium chrysogenum*	Not reported	[53
Chrysotriazole B (**50**)	“	“	“

Compound	Fungal producer	Other biological activities	Refs.
2-(4-Hydroxybenzoyl)-4(3*H*)-quinazolinone (**51**)	“	“	“
2-(4-Hydroxybenzyl)quinazolin 4(3*H*)-one (**52**)	“	“	“
*N*-[2-(4-Hydroxyphenyl)acetyl] formamide (**53**)	“	“	“
2-(4-Hydroxyphenyl)acetyl amide (**54**)	“	“	“
*N*-[(2*E*)-(4-Hydroxyphenyl) ethenyl]formamide (**55**)	“	“	“
*N*-[(2*Z*)-(4-Hydroxyphenyl) ethenyl]formamide (**56**)	“	“	“
Conio-azasterol (**57**)	*Coniothyrium cereale*	Low Antibiotic	[53]
*S*-Dehydroazasirosterol (**58**)	“	“	“
**4**,9-Dihydroxy-6-methyl-7- ((3-methylbut-2-en-1-yl)oxy)- 1H-phenalene-1,2,3-trione (**59**)	“	“	“
(*S*)-3,8-Dihydroxy-6-imino-1,9,9,10-tetramethyl-9,10-dihydrocyclohepta[4,5] naphtho[1,2-b]furan-5,7(4*H*,6*H*)-dione (**60**)	**“**	“	“
Deoxytrichodermaerin (**61**)	*Trichoderma longibrachiatum*	Inhibition of some plankton species	[54]
Harzianol A (**62**)	“	“	“
Harzianone (**63**)	“	“	“
Tetrahydroauroglaucin (**64**)	*Eurotium chevalieri*	Not reported	[55]
Dihydroauroglaucin (**65**)	“	“	“
Chrysin (**66**)	*Chaetomium globosum*	Antioxidative, antidiabetic, anti-inflammatory	[56]

“: identical data to the entry immediately above

**Fig. 1:**
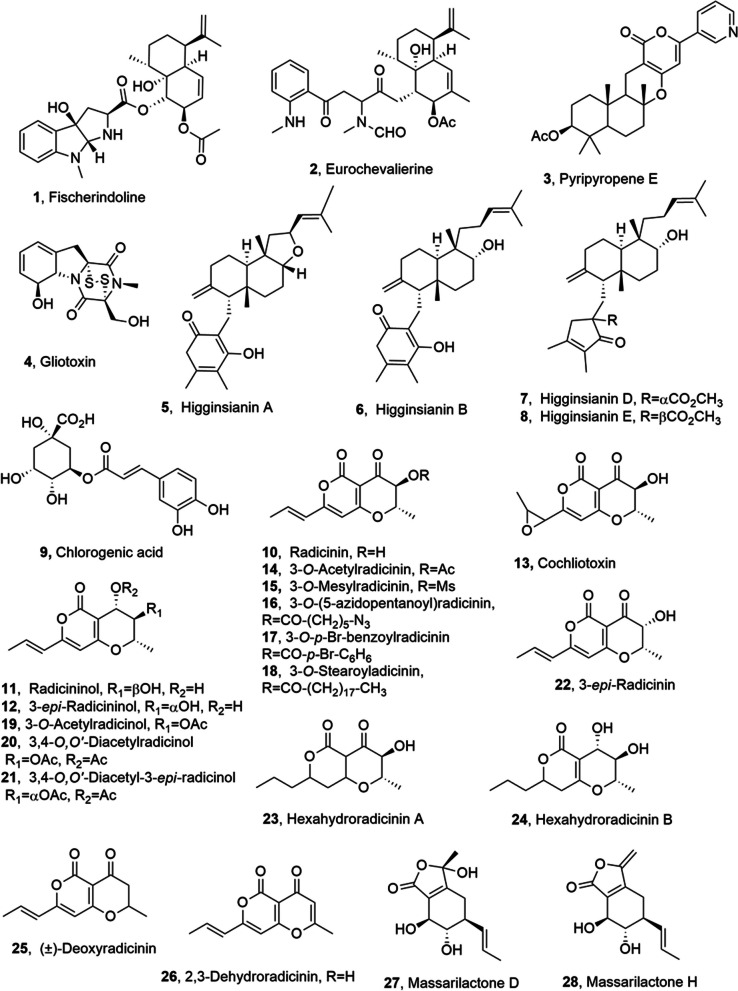
Metabolites isolated from terrestrial fungi

Fischerindoline (**1**, Fig. 1), a terpenoid pyrroloindole, was isolated from both solid and liquid cultures of *Neosartorya pseudofischeri*, together with eurochevalierine, pyripyropenes A and E, gliotoxin (**2**–**4**, Fig. 1), bis(dethio)bis(methylthio)gliotoxin and sequiterpene, [22]. *N. pseudofischeri* (Ascomycete) had been previously studied for the synthesis of secondary metabolites showing in vitro anticancer activity [23]. Fischerindoline (**1**) and the other metabolites were tested against a panel of six cancer cell lines, namely the five human cell lines as the A549 non-small cell lung cancer (NSCLC; DSMZ code ACC107), the SKMEL28 melanoma (ATCC code HTB-72), the Hs683 oligodendroglioma (ATCC code HTB-138), the U373 glioblastoma (ECACC code 89081403), the MCF7 breast cancer (DSMZ code ACC115) cell lines and the mouse cell line B16F10 melanoma (ATCC code CRL-6475). Fischerindoline (**1**) showed growth inhibitory effects against all the cancer cells tested and this activity was like to that exhibited by eurochevalierine (**2**) and pyripyropene E (**3**). Furthermore, all metabolites were much less potent than gliotoxin (**4**) [22]. Eurochevalierine (**2**) had been previously isolated together with chevalones A and D, aszonapyrone B, sequiterpene, a pyrrolobenzoxazine named CJ-12662, aszonapyrone A, and ergosterol from *Eurotium* *chevalieri*, showing in vitro cytostatic activity towards human U373 glioblastoma and A459 non-small-cell-lung cancer cells associated to a strong inhibition of mitotic rates [24]. Successively, in deep studies were carried out on eurochevalierine (**2**) mode of action in anticancer activity. Compound **2** inhibited sirtuins 1 and 2 activities (IC50 about 10 µM) without affecting sirtuin 3 activity. Sirtuins (NAD^+^-dependent histone deacetylases) are implicated in cellular processes such as proliferation, DNA repair, and apoptosis by regulating gene expression and the function of numerous proteins. Eurochevalierine (**2**) induces the acetylation of histone H4 and α-tubulin in various cancer cell models determining strong cytostatic activity without significantly affecting the viability of healthy PBMCs (Human peripheral blood mononuclear cells). Eurochevalierine preferentially targets cancer cell proliferation, as normal human primary CD34^+^ stem/progenitor cells were less affected by the treatment. Furthermore, eurochevalierine (**2**) showed like drug-likeness properties and therefore is a suitable scaffold as lead compound for optimization study on the mechanisms and biological roles of sirtuins and a potential base for therapeutic developments [25].

Higginsianins A and B (**5** and **6**, Fig. 1), which are two diterpenoid α-pyrones, were synthesized by *Colletotrichum higginsianum* in liquid culture [26]. Fungi belonging to *Colletotrichum* genus are among the most dangerous pathogens in agriculture being the responsible agents of serious diseases of many cultivated plants. This fungal genus is well known for its ability to synthesize a large number of secondary bioactive metabolites with potential application in different fields [27]. Higginsianins A and B (**5** and **6**) were assayed for antiproliferative activity against a panel of six cancer cell lines (five human cancer cell lines such as the Hs683 oligodendroglioma, the U373 glioblas-toma (GBM), A549 non-small-cell lung cancer (NSCLC), the MCF-7 breast carcinoma, and the SKMEL-28 melanoma models; murine cell line was the B16F10 melanoma model) and showed IC_50_ values, obtained with cells sensitive to proapoptotic stimuli, lower by more than 1 order of magnitude than their apoptosis-resistant cells (1 vs > 80 μM). The 22-*O*-acetyl and the 22-*O*-methyl derivatives of **5** and another its derivative showing the expansion of the furan ring into the corresponding pyran one were prepared and tested for antiproliferative activity. The two 22-*O*-modified derivatives exhibited IC_50_ values and differential sensitivity profiles like to those of **5**, that was lost when the furane ring was expanded in the pyran one [26]. Successively, the antiproliferative activity of **5** and **6** was more in deep investigated on the six above cited cancer cell lines in comparison with human primary keratinocytes and Hacat cells spontaneously immortalized, which are a preneoplastic cell line models. Compounds **5** and **6** reduced viability of A431, HeLa and H1299 cancer cells inducing the level increase of the cell cycle inhibitor p21WAF and the rate reduction of cell proliferation and caused the arrest cancer cells in S-phase. Furthermore, cells incubated with higginsianins induced DNA lesions, while increasing this times, both higginsianins induced notheworthy cell detachment and non-apoptotic cell death. These results showed that higginsianins exhibit significant cytotoxicity against a wide spectrum of malignant cancer cells and can be considered as potential anticancer agents [28].

Later, higginsianins D and E (**7** and **8**, Fig. 1), other two diterpenoids with a tetrasubstituted 3-oxodihydrofuran substituent, were obtained from the the same fungus namely *C. higginsianum*. The antiproliferative activity of metabolites **7** and **8** was tested against A431 cells derived from epidermoid carcinoma and H1299 non-small-cell lung carcinoma using HaCaT immortalized keratinocytes as a preneoplastic cell line model. Higgisianin E (**8**) exhibited higher cytotoxicity than higginsianin D (**7**), with an IC_50_ value of 1.0 μM against A431 cells while both compounds did not affected immortalized keratinocytes [29].

Chlorogenic acid (CGA, **9**, Fig. 1) was isolated from *Screlotium rolfsii*, which is a soil borne phytopathogen fungus with a wide host array [30]. CGA beloging to the family of hydroxyl cinnamic acid esters, is a polyphenol mostly present in vegetables and fruits. It was widely used in traditional Chinese medicine for various pharmacological activities such as antioxidant, antimicrobial, anti-inflammatory and hepato-protective activities etc. [31]. The effect of CGA (**9**) on the reversion of multidrug resistant (MDR) mediated by Pglycoprotein (P-gp) towards cancer cells was evaluated on human mdr1 gene, transfected mouse gene, transfected L5178 and L5178Y mouse T-cell lym-phoma. CGA was also estimated for antiproliferative activity on the L5178 mouse T cell lymphoma cell line. The results showed that CGA (**9**) possesses an excellent MDR reversing activity in a dose-dependent manner towards mouse T-lymphoma cell line and had antiproliferative effect on L5178Y mouse T-lymphoma cell line [32].

Non-ribosomal cyclodepsipeptides which include beauvericins (BEAs), enniatins (ENNs), and beauvenniatins (BEAEs) are one of the main mycotoxin groups. Lipodepsipeptides (LPDs) are a wide group of natural products spread among living organisms such as bacteria, fungi, yeasts, virus, insects, plants and marine organisms and contain a lipid connected to a peptide, which are able to self-assemble into several different structures [33]. BEAs, ENNs and BEAEs were not only produced by fungi belonging *Fusarium* genus but also from other fungal genera such as *Beauveria*, *Acremonium* and *Paecilomyces*. Due to their mycotoxic activity they may be very azardous for human. However, these cyclodepsipeptides can help the development of new drug considering their antimicrobial, insecticidal, antifungal, and antibiotic activities, as well as, their cytotoxicity suggesting their possible applications in anticancer therapy [34].

Radicinin (**10**, Fig. 1), a dihydropyranopyran-4,5-dione, was isolated from *Cochlobolus australiensis* together with radicinol and its 3-epimer and cochliotoxin, which is a radicinin epoxy analogue (**11**–**13**, Fig. 1). The fungus was proposed as a mycoherbicide to control buffelgrass (*Pennisetum ciliare* or *Cenchrus ciliaris*), which is a perennial grass that has become highly invasive in the Sonoran Desert of southern Arizona. In fact, buffelgrass caused severe damage by competition for water and nutrients with native plant species [35]. Radicinin showed a target-specific phytotoxicity towards the host plant and no toxicity on zebrafish embryos, demostrating its potential to develop a natural herbicide to manage buffelgrass. On the basis of these results a SAR study was performed using the natural analogues of radicinin and preparing some key hemisynthetic derivatives as its acetyl, mesyl, 5-azidopentanoyl, *p*-bromobenzoyl and stearoyl esters (**14**–**18**, Fig. 1) and the mono- and di-acetyl ester of radicinol, its 3 epimer and 3-e*pi*-radicinin (**19**–**22**, Fig. 1), and two hexahydro derivatives of radicinin A and B (**23** and **24**, Fig. 1). Leaf puncture bioassay was used to test the phytotoxic activity towards buffelgrass. Most of the compounds showed lower phytotoxicity than radicinin. The α,β-unsaturated carbonyl group at C-4, as well as, a free secondary hydroxy group at C-3 and its stereochemistry proved to be important features to impart activity [35]. These results suggested to develop a total ensatioselective synthesis of radicinin or that of some its active analogues. In particular, the synthesis of racemic (±)-3-deoxyradicinin (**25**, Fig. 1), which is the immediate biosynthetic precursor of radicinin (**10**) [36] was realized and aimed to determine the role of the hydroxygroup at C-3 and its absolute configuration to impart phytotoxicity [37]. The results of (±)-3-deoxyradicinin (**25**) assay, suggested that the stereochemistry of C-3 seemed to have some role, because 3-*epi*-radicinin was less active [38]. Successively, radicinin (**10**) was tested for its anticancer activity towards three human cancer cell lines such as A549 NSCLC (DSMZ code ACC107), Hs683 oligodendroglioma (ATCC code HTB-138) and SKMEL-28 melanoma (ATCC code HTB-72) cells, and showed a promising toxicity (IC_50_ values of 7.7 ± 0.6, 8.7 ± 0.4, 8.2 ± 0.2 μM). These results suggested to perform a SAR study employing natural and synthetic derivatives of radicinin such as radicinol, and its 3-epimer, the 3-*O*-acetyl, 3-*O*-mesyl and 3-*O*-(5-azidopentanoyl) esters of radicinin, 3,4-*O,Oʹ*-diacetylradicinol ( ±)-3-deoxyradicinin (**11** and **12**, **14**–**16**, **20** and **25**) and 2,3-dehydroradicinin (**26**, Fig. 1) together with the four synthetic intermediates 4-methoxy-6-methyl-2*H*-pyran-2-one, 3-bromo-4-methoxy-6-methyl-2*H*-pyran-2-one, (*E*)-4-methoxy-6-(propen-1-yl)-2*H*-pyran-2-one and (*E*)-3-bromo-4-methoxy-6-(propen-1-yl)-2*H*-pyran-2-one. The anticancer activity was tested towards the same three cancer cell lines above cited. Radicinol (**11**), its 3-epimer (**12**) and the corresponding 3,4-*O,Oʹ*-diacetyl derivative (**20)** had no activity showing that the carbonyl at C-4 is an important structural feature to impart anticancer activity. This is due to its capacity to allow a Michael addition of a nucleophile residue. The activities of (±)-3-deoxy- and 2,3-dehydroradicinin (**25** and **26**) were slightly lesser than that of radicinin (**10**), demonstrating that the 3-hydroxy group plays a minor role in the activity. Among the synthetic intermediates only the methoxypyrones showed moderate anticancer activities [38]. The results of this SAR study are in good agreement with those obtained testing the phytotoxic activity against the host plant and other infestant plants also using other natural analogues of **10** [35]. Furthermore, any difference in the activity towards the three cancer cell lines was observed suggesting that the in vitro, the anticancer activity of radicinin could occurs through non-apoptotic pathways and thus it represents a further potential to combat chemoresistant cancers [38]. The low yield of radicinin obtained from the fungal fermentation, unfortunately limit its direct use in clinical trials but a more practical alternative could be (±)-3-deoxyradicinin (**25**), which may be obtained in a straightforward way through a novel synthetic strategy [37].

Massarilactones D and H (**27** and **28** Fig. 1), which are two polyketides, were obtained from *Kalmusia variispora*, which is a fungus associated with grapevine trunk diseases (GTDs). The fungus was identified in Iran and caused in greenhouse conditions serious symptoms on the host plant. Both furanones **27** and **28**, at variable concentrations and depending on the day of inoculation, showed phytotoxicity on *Vitis vinifera* L. [39]. Massarilactones D and H were tested for their anticancer activity against A549 non-small cell carcinoma (NSCLC), Hs683 oligodendroglioma and SKMEL-28 melanoma cells. While massarilactone D (**27**) was inactive on all the cancer cell lines, masssarilactone H (**28**) showed a good cytotoxicity (IC_50_ values of 32.9 ± 3.5, 31.6 ± 2.5, 35.2 ± 2.8 μM, respectively). These results suggest that the exomethylene group present in the compound **28** and not in **2**7 may be an important structural feature for the activity [39]. However, in a previous study and when tested at 1.3 mM against KB-3–1 cervix cancer cell line and L929 mouse fibroblasts both compounds **27** and **28** were inactive. Of course, the difference in cancer type might explain this contrasting results [40].

## Metabolites from endophytic fungi

Among microorganisms which spend all or part of their lifecycle inter- and/or intra-cellarlluy colonizing healthy tissues of their host there are the endophytic plant fungi. They, typically, not caued apparent disease symptoms and their relationship with host may range from latent pathogenesis to mutualistic symbios. Since the first endophytic fungus discovered in darnel on 1904, renewed attention was paid to the chemistry and bioactivity of endophytic fungi metabolites and their potential practical application in different fields [41]. This section is focused on metabolites with anticancer activity and it is divided in two subsections regarding the metabolites produced from terrestrial and marine endophytic fungi, respectively (Table 2).

### Metabolites from endophytic terrestrial fungi

**Fig. 2:**
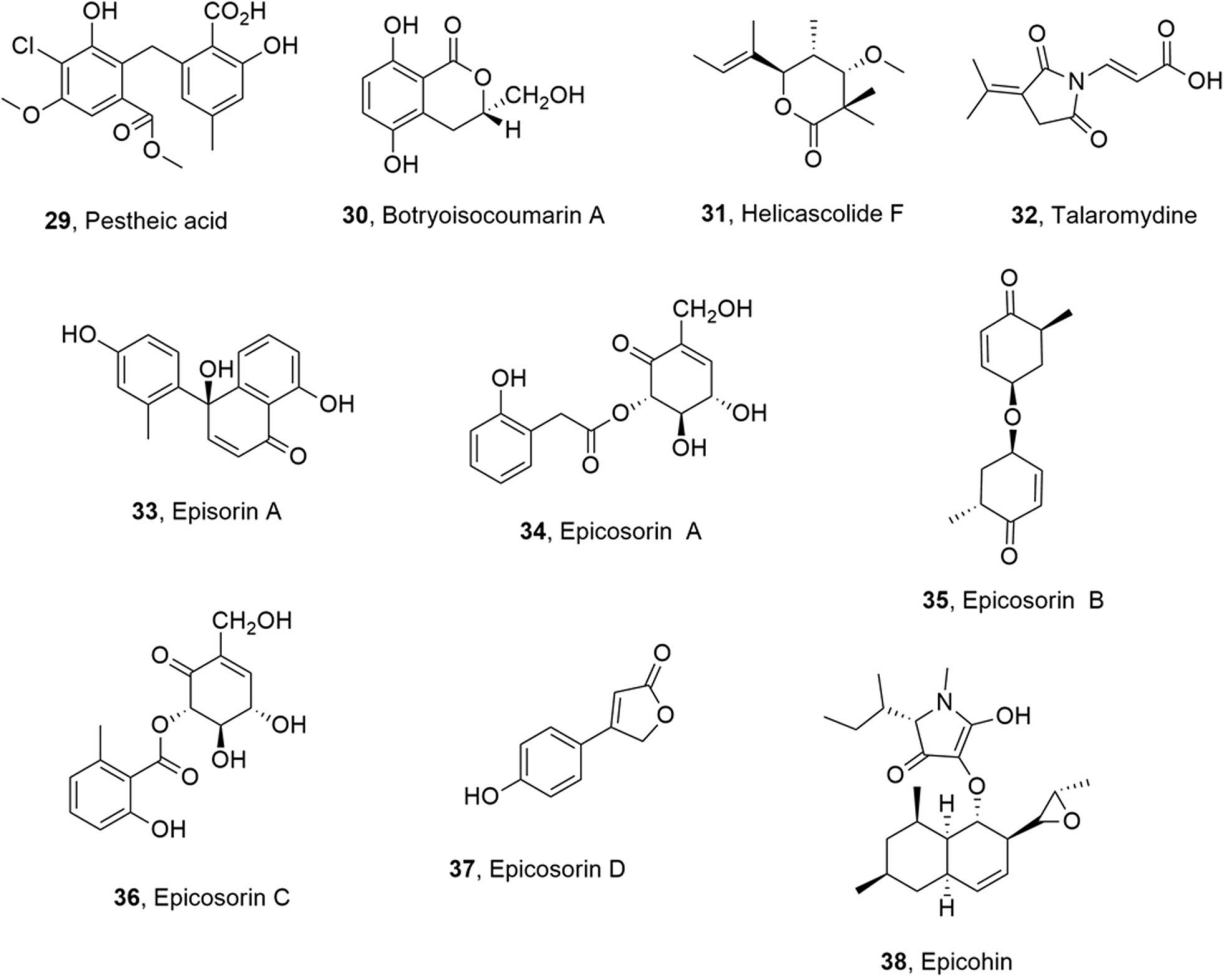
Metabolite isolated from endophytic terrestrial fungi

Pestheic acid, or dihidromaldoxin, (**29**, Fig. 2), which is a chlorinated diphenylic ether, was produced from the fungus *Pestalotiopsis guepinii*. This fungus lives associated to *Virola michelii*, which is a plant widely diffused in the Amazon rain-forest. This plant was also known as “ucuúba preta”, whose leaves are used by the indi-geneous people as a medicine to ease fungal skin rashes [42]. Compound **29**, which is know as a plant growth regulator [43], was isolated in 1996 [44]. The cytotoxic, cytostatic, and genotoxic activity of pestheic acid (**29**) was tested on gastric adenocarcinoma cell line (PG100) and clonogenic acid survival decreased. Compound **29** also caused significant increases in both micronucleus and nucleoplasmic bridge frequency. However, changes were not observed in cell cycle kinetics or apoptosis induction, while the genotoxicity and mutagenicity of compound **29** can justify the induction of reactive oxygen species. The lack in PG100 cell line of the TP53 gene, which is common in gastric cancers, could determine the absence of repair checkpoints. Pestheic acid (**29**) showed clear cytotoxic effect but only at high concentration with a IC_50_ of 50.5 μg/mL, which demonstrated that it is not a potent anticancer compound [45].

Botryoisocoumarin A (**30**, Fig. 2), a 3*S*-5,8-dihydroxy-3-hydroxymethyldihydroisocoumarin, was isolated together with monocerin, 3-methyl-6,8-dihydroxyisocoumarin, 8-methoxymellein, *trans*-4-hydroxymellein and 5-hydroxy-7-methoxy-4,6-dimethyl phthalide from a solid culture of *Botryosphaeria* sp. KcF6, an endophytic fungus associated to the mangrove plant *Kandelia candel* [46]. All the compounds were assayed for their cytotoxic and antinflammatory (COX-2) activities. Ten human tumor cell lines, namely K562, MCF-7, A549, U937, HeLa, DU145, HL60, BGC823, MOLT-4 and H1975 were used, according to Bergeron et al. (1984) to test the cytotoxicity [47]. A strong COX-2 inhibitory activity with an IC_50_ value of 6.51 μM was showed testing botryoisocoumarin A (**30**), whereas none of the compounds exhibited cytotoxicity on the tested cancer cell lines (IC_50_ > 100 μM) [46].

Helicascolide F and talaromydine (**31** and **32** Fig. 2), a lactone and a pyrrolidine derivative, repectively, were isolated together with (*E*)-3-(2,5-dioxo-3-(propan-2-ylidene) pyrrolidin-1-yl)acrylic acid, 2-deoxyribonolactone, pyranonigrin S, pyranonigrin A, 3,4-dihydroxyphenylacetic acid methyl ester and 3,4-dihydroxybenzeneacetic acid from *Talaromyces assiutensis* JTY2, which is an endophytic fungus obtained from the *Ceriops tagal* leaves collected in the South China Sea. All compounds were assayed for cytotoxic activities against HeLa, MCF-7 and A549 cancer cell lines and only helicascolide F and talaromydine (**31** and **32**) and (*E*)-3-(2,5-dioxo-3-(propan-2-ylidene) pyrrolidin-1-yl)acrylic acid exhibited moderate inhibition of HeLa, MCF-7 and A549 cells with (IC_50_ values ranging from 14.1 to 38.6 μM). Only pyranonigrin A, the 3,4-dihydroxyphenylacetic acid methyl ester and the 3,4-dihydroxybenzeneacetic acid, when tested against six phytopathogenic fungi such as *Alternaria brassicicola*, *Phytophthora parasitica* var. *nicotianae*, *Colletotrichum capsici*, *Bipolaris oryzae*, *Diaporthe medusaea nitschke* and *Ceratocystis paradoxa moreau*, showed a broad antifungal activity spectrum [48].

Episorin A, epicosorins A-D and epicohin (**33**, **34**–**37** and **38**, Fig. 2) were isolated together with 2,5-dihydroxybenzene methanol methyl ether, scytalone, chlorogentisyl alcohol (2*S*,4*S*,5*R*)-4,5-dihydroxy-2-methylcyclohexanone, epoxydon, 2,5-dihydroxybenzaldehyde, 3-hydroxybenzyl alcohol and gentisyl alcohol from *Epicoccum sorghinum* grown on *Thelephora ganbajun* medium, which is the host mushroom. Most of the compounds showed at concentration of 50 μg/cm^2^ antifeedant activity towards silkworm with feeding deterrence index (FDI) of 50–99%, The fungal organic extract and episorin A, epicosorinA exhibited the higher antifeedant activity. These results indicated that the interaction between *E. sorghinum* with *T. ganbajun* medium determined more resistance to pest and prompt the synthesis of novel antibiotics. Episorin A (**33**) had highest antifungal activities against *Trichoderma harzianum*, *Pithomyces chartarum*, and *Penicillium ochrochloron*. All compounds showed antifungal activity against at least one fungus with MICs ≤ 32 μg/mL. Episorin A and epicosorinA (**33** and **34**) did not showed NO inhibition and anti-acetylcholinesterase activities but the first one (**33**) had a significant inhibition on NO production in LPS-activated macrophages (IC_50_ values of 5.40 ± 0.25 μM) with an effect higher than that of positive drug as NG-monomethyl-l-arginine (l-NMMA), cytotoxicity towards HL-60, A-549, SMMC-7721, MCF-7 and SW480 (IC_50_ a 14.21 ± 0.531–28.36 ± 0.43 μM), and obviously anti-acetylcholinesterase with IC_50_ at 4.32 μM [49].

### Metabolites from endophytic marine fungi

**Fig. 3:**
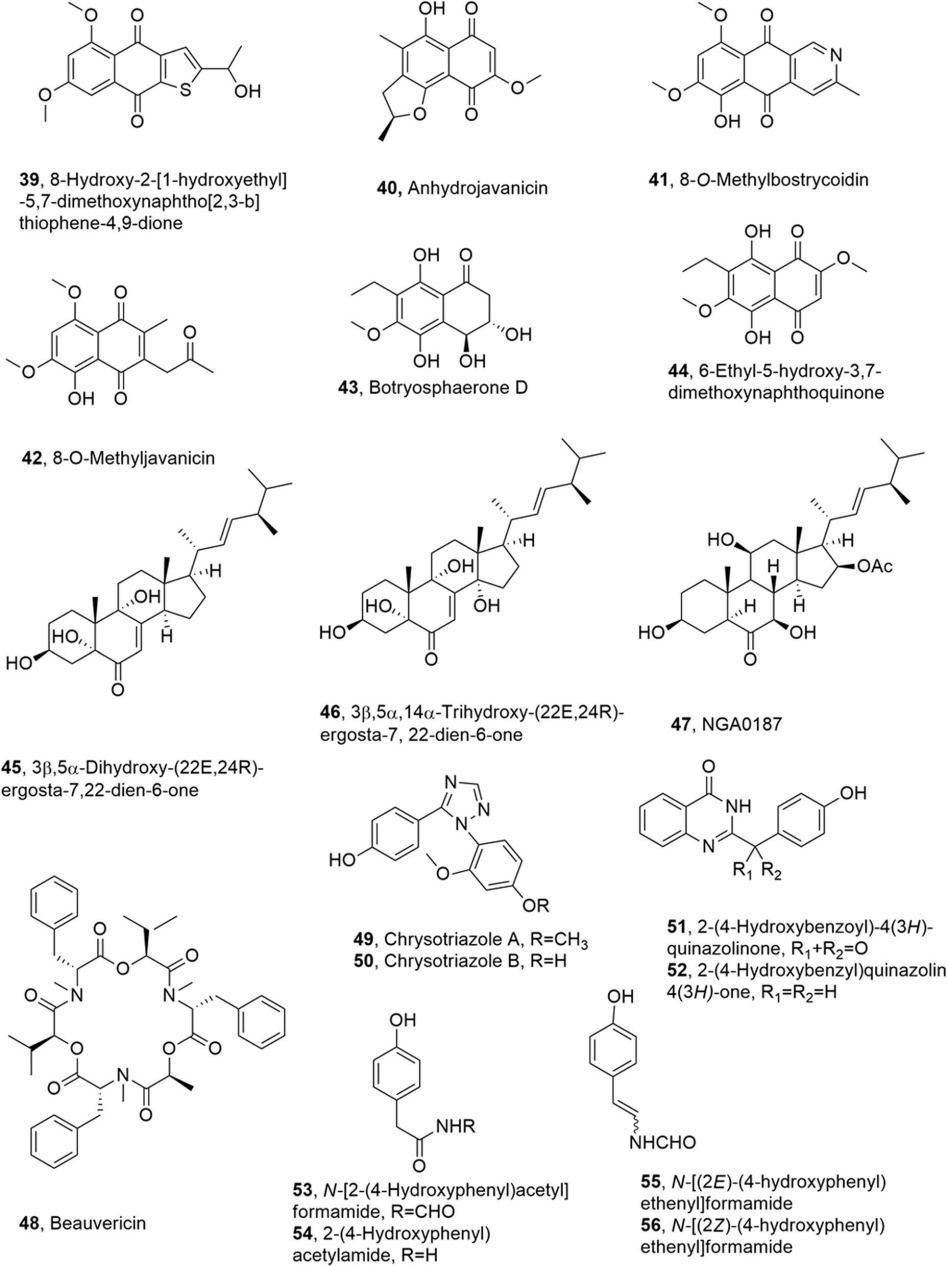
Metabolites isolated from endophytic marine fungi

8-hydroxy-2-[1-hydroxyethyl]-5,7-dimethoxynaphtho[2,3-b] thiophene-4,9-dione, a rare thiophene compound, was obtained from *Aspergillus terreus* (No. GX7-3B), a mangrove endophytic fungus, together with anhydrojavanicin, 8-*O*-methylbostrycoidin, 8-*O*-methyljavanicin, botryosphaerone D, 6-ethyl-5-hydroxy-3,7-dimethoxynaphthoquinone, 3β,5α-dihydroxy-(22*E*,24*R*)-ergosta-722-dien-6-one, 3β,5α,14α-trihydroxy-(22*E*,24*R*)-ergosta-7,22-dien-6-one, NGA0187 and beauvericin (**39**–**48**, Fig. 3) The fungus was isolated from a branch of *Bruguiera gymnoihiza* (Linn.) Savigny, which grows in the coastal salt marsh of the South China Sea in Guangxi province [50]. Compounds **40**, **41**, **47** and **48** showed a strong inhibition towards α-acetylcholinesterase (AChE) (IC_50_ values 2.01, 6.71, 1.89, and 3.09 μM, respectively). Furthermore, compounds **45** and **48** showed strong or moderate cytotoxicity towards MCF-7, A549, Hela and KB cell lines (IC_50_ values 4.98 and 2.02 (MCF-7), 1.95 and 0.82 (A549), 0.68 and 1.14 (Hela), and 1.50 and 1.10 μM (KB), respectively). Compound **46** induced weak growth inhibition of these tumor cell lines. The other metabolites had not cytotoxic activity [50]. Compound NGA0187 (**47**) was also previously obtained from *Acremonium* sp., which was isolated from decayed leaf sample collected in Nagoshi, Okinawa Prefecture, Japan. NGA0187 caused significant neurite outgrowth in PCl2 cells, but it is not observed the survival effect of NGA0187 on the primary culture of cerebral cortical neurons [51].

Chrysotriazoles A and B (**49** and **50**, Fig. 3), which are two triazoles, were obtained from *Penicillium chrysogenum* E-N118 along with 2-(4-hydroxybenzoyl)-4(3H)-quinazolinone, 2-(4-hydroxybenzyl)quinazolin-4(3*H*)-one, *N*-[2-(4-hydroxyphenyl)acetyl]formamide, 2-(4-hydroxyphenyl)acetylamide, *N*-[(2*E*)-(4-hydroxyphenyl)ethenyl]formamide and *N*-[(2*Z*)-(4-hydroxyphenyl)ethenyl]formamide (**51**–**56**, Fig. 3). The endophytic fungus was collected from the marine brown alga *Sargassum palladium*. Compounds **52**, **53**, and **55** showed moderate cytotoxicity towards Du145, A-549, and HeLa cell lines, with IC_50_ values of 8, 20, and 20 μg/mL, respectively [52].

**Fig. 4:**
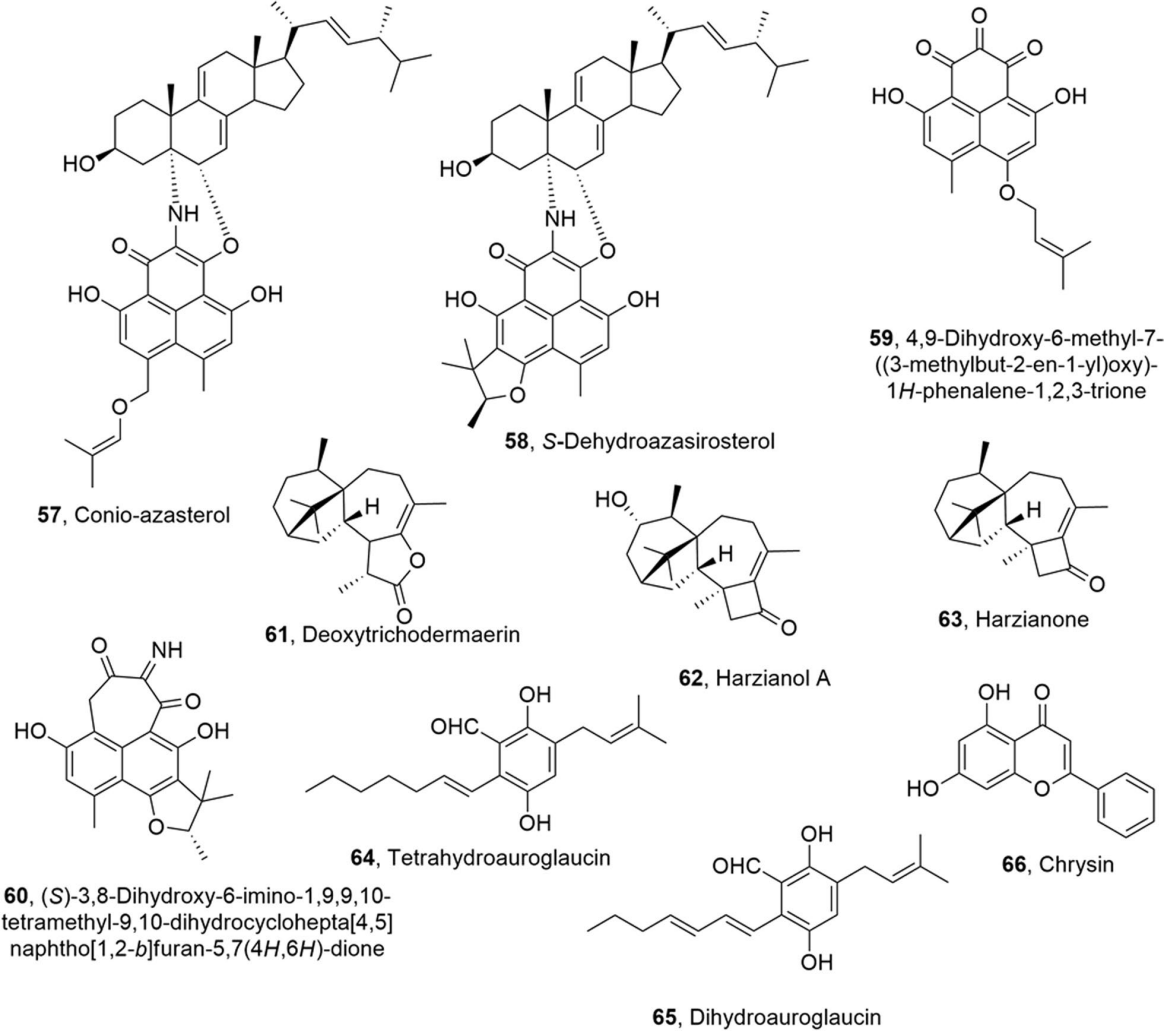
Metabolites isolated from endophytic marine fungi

Conioazasterol and *S*-dehydroazasirosterol (**57** and **58**, Fig. 4), which are phenalenone metabolites, and other two close metabolites (**59** and **60**, Fig. 4) were isolated together with ergostane-type sterol and entatrovenetinone from *Coniothyrium cereale*, which is a marine-derived endophytic fungus collected from the Baltic Sea algae *Enteromorpha* sp. The antimicrobial and cytotoxic activity of the isolated compounds was tested and they showed weak antibacterial activity against *Staphylococcus aureus*, *Escherichia coli*, *Pseudomonas aeruginosa*, and *Candida albicans*. Compounds **57** and **58**, the acetone adducts of entatrovenetinone, compound **59** and the hydrated derivative of compound **60** were assayed for cytotoxicity towards SKM1, U266, and K562 cancer cell lines. Only the derivative of compounds **60** had moderate cytotoxic activity (IC_50_ values ranging between 75, 45, and 8.5 µM against SKM1, U266, and K562 cancer cell lines, respectively). Furthermore, compounds **57** and **58** were object of molecular docking studies on estrogen receptor α-ligand binding domain (ERα-LBD). This study aimed to correlate their anti-proliferative activity with binding energies and affinities calculated from molecular docking [53].

Deoxytrichodermaerin (**61**, Fig. 4), which is a harziane lactone, was isolated from *Trichoderma longibrachiatum* together with harzianol A and harzianone (**62** and **63**, Fig. 4) A-WH-20–2, a fungal endophyte associated to the marine red alga *Laurencia okamurai*. All the metabolites showed a strong inhibition of some marine plankton species [54].

Tetrahydroauroglaucin (TAG) and dihydroauroglaucin (DAG) (**64** and **65**, Fig. 4) were isolated from the fungal endophyte *Eurotium chevalieri* MUT 2316, which was obtained from the Mediterranean sponge *Granti*a *compressa*. The compounds **64** and **65** had strong efficacy to inhibit human neuroblastoma SH-SY5Y cell migration. In particular, DAG (**65**) showed a marked inhibition of the highly migratory phenotype of SH-SY5Y cells, whereas TAG (64), no exhibited activity [55].

Chrysin (**66**, Fig. 4), which is a natural dihydroxyflavone, showed different biological activities including anticancer, antioxidative, antidiabetic, anti-inflammatory activity etc. Unfortunately, chrysin was extract in very low yield from honey plants, and this procedure is not scalable, not convenient and affected by several factors, such as geography, season and climatic conditions, which noteworthy decrease the production of compound **66**. An efficacy alternative is the microbial production of chrysin by the fungal marine endophyte *Chaetomium globosum*, obtained from a marine green alga. During this fermentation, also other flavanoids were produced including dihydrokaempferol, chalcone, galangin, baicalein. Threfore, three different strategies were applayied to enhance the production of chrysin: (i) optimization of growth medium, incubation time, pH, and temperature; (ii) the addition of biosynthetic flavonoid intermediates, i.e., phenylalanine and cinnamic acid; (iii) elicitation with polysaccharide, yeast extract and antibiotic and other elicitors that include UV radiation, salinity, and metal stress. The optimization of these parameters determine a 97-fold increase in the chrysin yield [56]

## Metabolites from marine fungi

Several metabolites with anticancer activity and belonging to different class of natural compound were isolated from marine fungi (Table 3).

**Table 3:** Metabolites with anticancer activity from marine fungi

Compound	Fungal producer	Other biological activities	Refs
(1′*S*)-7-Chloroaverantin (**67**)	*Aspergillus*	Not reported	[62]
(1′S)-6-*O*-Methyl-7-chloroaverantin (**68**)	“	“	“
(1′*S*)-1′-*O*-Methyl-7-chloroaverantin (**69**)	“	“	“
(1′*S*)-7-chloroaverantin,	“	“	“
6,1′-O,O-Dimethyl-7-chloroaverantin (**70**)	“	“	“
(1′*S*)-7-Chloroaverantin-1′-butyl ether (**71**)	“	“	“
7-Chloroaverythrin (**72**)	“	“	“
6-*O*-Methyl-7-chloroaverythrin (**73**)	“	“	“
(1′*S*)-6,1′-*O,O'*-Dimethyl-7-bromoaverantin (**74**)	“	“	“
(1′S)-6-*O*-Methyl-7-bromoaverantin (**75**)	“	“	“
(1′S)-6,1′-*O,O'*-Dimethylaverantin (**76**)	“	“	“
Chaetomugilin S (**77)**	*Chaetomium globosum*	“	[63]
Dechloto-chaetomugilin A (**78)**	“	“	“
Dechloro-chaetomugilin D (**79)**	“	“	“
*Bis*(dethio)-10a-methylthio-3a-deoxy3,3a-didehydrogliotoxin (**80**)	*Penicillium* sp.	“	[64]
6-Deoxy-5a,6-didehydro gliotoxin (**81**)	“	“	“
Gliotoxin (**82**)	“	HMT G9a inhibitory activity	“
*Bis*(dethio)bis(methylthio)gliotoxin (**83**)	“	Not reported	“
*Bis*(dethio)*bis*-(methylthio)-5a,6-didehydrogliotoxin, (**84**)	“	“	“
5a,6-Didehydrogliotoxin, (**85**)	“	HMT G9a inhibitory activity	“
Gliotoxin G (**86**)	“	“	“
Aspergiolide A (**87**)	*Aspergillus glaucus*	Not reported	[65, 66]
Auranomide A (**88**)	*Penicillium aurantiogriseum*	**“**	[67]
Auranomide B (**89**)	“	“	“
Auranomide C (**90**)	“	“	“
Penicacid A (**91**)	*Penicillium* sp.	Inhibition of IMPDH Immunosuppresive	[68]
Penicacid B (**92**)	“	Inhibition of IMPDH	“
Penicacid C (**93**)	“	Inhibition of IMPDH Immunosuppresive	“
6-*O*-Desmethyldechlorogriseofulvin (**94**)	*Nigrospora* sp.	Not reported	[72]
6’-Hydroxygriseofulvin (**95**)	“	“	“
2,3-Didehydro-19a-hydroxy-14-epicochlioquinone B **(96**)	“	Antibacterial	“
Fumiquinazoline K (**97**)	*Aspergillus fumigatus*	Not reported	[74]
6β,16β-Diacetoxy-25-hydroxy-3,7-dioxo-29-nordammara-1,17(20)-dien-21,24-lactone (**98**)	“	“	**“**
(1*S*,*2S*,3*S*,5a*S*,10a*R*)-1,10a-Dihydroxy-6'-methoxy-3- (2-methylprop-1-en-1-yl)-5a,6,7,8-tetrahydro-1*H*-spiro[dipyrrolo[1,2-a:1',2'-d]pyrazine-2,2'-indoline]-3',5,10(3*H*,10a*H*)-trione (**99**)	“	Moderate cytotoxicity	**“**
Fischeacid (**100**)	*Neosartorya fischeri*	Not reported	[75]
Fischexanthone (**101**)	“	“	“
Expansol C (**102**)	*Penicillium expansum*	Cytotoxic	[76]
Expansol D (**103**)	“	Not reported	“
Expansol E (**104**)	“	Cytotoxic	“
Expansol F (**105**)	“	Not reported	“
3-*O*-Methyldiorcinol (**106**)	“	“	“
Toluquinol (**107**)	*Penicilium sp.*	**“**	[77]
JBIR-27 (**108**)	*Penicillium citrinum*	“	[78]
Petasol (**109**)	“	“	“
AO-1 (**110**)	“	Cytotoxic Anti yeast	“
Dihydro-AO-1 (**111**)	“	Cytotoxic	“
Aculeatusquinone A (**112**)	*Aspergillus aculeatus*	“	[79]
Aculeatusquinone B (**113**)	“	Not reported	“
Aculeatusquinone C (**114**)	“	“	“
Aculeatusquinone D (**115**)	“	Cytotoxic	“
Penitrem A (**116**)	*Penicillium commune*	Nematocidal	[80]
Penitrem B (**117**)	“	Not reported	“
Penitrem C (**118**)	“	“	“
Penitrem D (**119**)	“	“	“
Penitrem E (**120**)	“	“	“
Penitrem F (**121**)	“	“	“
Paspaline (**122**)	“	“	“
Emnidole SB (**123**)	“	“	“
6-Bromopenitrem B (**124**)	“	“	“
6-Bromopenitrem E (**125**)	“	Nematocidal	“
Penicitrinone E (**126**)	*Penicillium sp.*	Not reported	[81]
Penicitrinol J (**127**)	*“*	Antibiotic	“
Penicitrinol K (**128**)	“	“	“
Citrinolactone D (**129**)	**“**	Not reported	“
2-(4-Hydroxybenzoyl) quinazolin-4(3*H*)-one (**130**)	*Penicillium oxalicum*	“	[82]
7,8-Dihydroxy-3,5,7-trimethyl-8,8a-dihydro-1*H*-isochromen-6(7*H*)-one (**131)**	*Eutypella scoparia*	“	[83]
6-(Hydroxymethyl)-2,2-dimethyl-3,4-dihydro-2*H*-chromene-3,4-diol (**132**)	“	“	“
Dankastatin C (**133**)	*Gymnascella dankaliensis*	“	[87]
Aszonalenin analogue 1c (**136**)	*Neosartorya fischeri*	“	[88]
Sartorypyrone A (**137**)	“	“	“
Aszonalenin (**138**)	“	“	“
Acetylaszonalenin (**139**)	“	“	“
13-Oxofumitremorgin B (**140**)	“	“	“
Aszonapyrone A (**141**)	*Neosartorya fischeri* *Neosartorya laciniosa*	“	“
Helvolic acid (**142**)	*Neosartorya fischeri* *Neosartorya tsunodae*	“	**“**
Aszonapyrone B (**143**)	*Neosartorya laciniosa*	“	“
Tryptoquivaline L (**144**)	“	“	“
3ʹ-(4-Oxoquinazolin-3-yl)spiro[1*H*-indole-3,5ʹ-oxolane]-2,2ʹ-dione (**145**)	“	“	“
Sartorypyrone B (**146**)	“	“	“
Trichoderiol C (**147)**-	*Trichoderma citrinoviride*	“	[89]
Citrinoviric acid (**148)**-	“	“	“
Penicillenol D (**149)**-	“	“	“
(+)-6-*O*-Demethyl pestalotiopsin A (**150**)	*Ascotricha* sp.	“	[90]
(+)-6-*O-*Demethyl Pestalotiopsin C (**151**)	“	“	“
(−)-6-*O*-Demethyl Pestalotiopsin B (**152**)	“	“	“
Penicimutalide A (**153**)	*Penicillium purpurogenum*	“	[91]
Penicimutalide B (**154**)	“	“	“
Penicimutalide C (**155**)	“	“	“
Penicimutalide D (**156**)	“	“	“
Penicimutalide E (**157**)	“	“	“
Penicimutalide F (**158**)	“	“	“
Penicimutalide G (**159**)	“	“	“
Chondrosterin I (**160**)	*Chondrostereum* sp.	“	[92]
Chondrosterin J (**161**)	“	“	“
Scopularide A (**162**)	*Scopulariopsis brevicaulis*	“	4
Scopularide B (**163**)	“	“	**“**
Pseurotin A (**164**)	*Aspergillus* sp.	“	[94]
Pseurotin D (**165**)	“	“	“
Pseurotin FD-838 (**166**)	“	“	“
Fumitremorgin C (**167**)	“	“	“
12,13-Dihydroxy fumitremorgin C (**168**)	“	“	“
Methylsulochrin (**169**)	“	“	“
Bis(dethio)bis(methylthio) gliotoxin (**170**)	“	“	“
Curvularin (**171**)	*Penicillium purpurogenum*	“	[95]
Citrinin (**172**)	“	“	“
Penicitrinone (**173**)	“	“	“
*erythro*-23-*O*-Methyl neocyclocitrinol (**174**)	“	“	“
22*E*-7α-Methoxy-5α,6α-epoxyergosta-8(14),22-dien-3β-ol (**175**)	“	“	“
Rhizovarin C (**178**)	“	“	“
Rhizovarin D (**179**)	“	“	“
Rhizovarin E (**180**)	“	“	“
Rhizovarin F (**181**)	“	“	“
Aurasperone A (**182**)	*Aspergillus niger*	“	[98]
Fonsecinone D (**183**)	“	“	“
Aurasperone F (**184**)	“	COX-2–inhibition	“
Fonsecinone B (**185**)	“	Not reported	“
Aurasperone B (**186**)	“	“	“
Aurasperone C (**187**)	“	COX-2–inhibition	“
Fonsecinone A (**188**)	“	Not reported	“
Asperpyrone A (**189**)	“	COX-2–inhibition	“
Fonsecinone C (**190**)	“	Not reported	“
Asperpyrone D (**191**)	“	“	“
Asperpyrone E (**192**)	“	“	“
2,4-Dihydroxy-3-methylacetophenon (**193**)	*Neosartorya siamensis*	“	[99]
Chevalone C (**194**)	“	“	“
Nortryptoquivaline (**195**)	“	“	“
Tryptoquivaline H (**196**)	“	“	“
Tryptoquivaline F (**197**)	“	“	“
Fiscalin A (**198**)	“	“	“
*epi*-Fiscalin A (**199**)	“	“	“
*epi-*Neofiscalin A (**200**)	“	“	“
*epi*-Fiscalin C (**201**)	“	“	“
Phomoxanthone A (**202**)	*Phomopsis longicolla*	“	[100]
Asperphenin A (**203**)	*Aspergillus* sp.	“	[101]
Asperphenin B (**204**)	“	“	“
Demethoxyfumitremorgin C (**205**)	*Aspergillus fumigatis*	“	[103]
Secalonic acid H (**206**)	*Penicillum oxalicum*	“	[104]
Secalonic acid I (**207**)	“	“	“
Secalonic acids D (**208**)	“	“	“
Dichotone A (**209**)	*Dichotomomyces* sp.	“	[105]
Dichotone B (**210**)	“	“	“
Diorcinolic acid **(211**)	*Aspergillus sydowii*	“	[106]
β-d-Glucopyranosyl aspergillusene A (**212**)	“	“	“
Tolypocladenol C (**213**)	*Tolypocladium geodes*	“	[107]
Penicimutanolone A (**214**)	*Penicillium purpurogenum*	“	[108]
Penicimutanolone B (**215**)	“	“	“
Penicimutanolone A methyl ether (**216**)	“	“	“
Penicimumide (**217**)	“	“	“
Asperindole A (**218**)-	*Aspergillus* sp.	“	[109]
Asperindole B (**219**)-	“	“	“
Asperindole C (**220**)-	“	“	“
Asperindole D (**221**)-	“	“	“
26-Membered polyene macrolactam (**222**)	*Micromonospora* sp.	“	[110]
Peniciphenalenin A (**223**)	*Penicillium* sp.	“	[111]
Peniciphenalenin B (**224**)	“	“	“
Peniciphenalenin E (**227**)	“	“	“
Peniciphenalenin F (**228**)	“	“	“
Asperchalasine A (**229**)	*Aspergillus flavipes*	Antiangiogenic	[112]
Lithocarpinol A (**230**)	*Phomopsis lithocarpus*	“	[113]
Lithocarpinol B (**231**)	“	“	“
Porosuphenol A (**232**)	*Aspergillus porosus*	Not reported	[114]
Porosuphenol B (**233**)	“	“	“
Porosuphenol C (**234**)	“	“	“
Porosuphenol D (**235**)	“	“	“
Gentisyl alcohol (**236**)	*Arthrinium* sp.	“	[115]
Emerixanthone E (**237**)	*Emericella* sp.	Antibiotic No anticancer	[116]
Penicimutanin A (**238**)	*Penicillium purpurogenum*	Not reported	[117]
Penicimutanin B (**239**)	**“**	“	**“**
Hypoxone A (**240**)	*Hypoxylon rubiginosum*	“	[118]
(3*S*,6*S*)-3,6-Dibenzylpiperazine-2,5-dione (**241**)	*Paecilomyces formous*	“	[119]
2-(2ʹ,3-epoxy-1ʹ,3ʹ,5ʹ-heptatrienyl)-6-hydroxy-5-(3-methyl-2-butenyl) benzaldehyde (**242**)	*Aspergilus sp.*	“	[120]
(−)-(3*R*,6*R*)-Hyalodendrin (**243**,	*Paradendryphiella salina*	“	[121]
(−)-(3*R*,6*R*)*bis*-Dethiodi(methylthio)hyalodendrin (**244**)	“	“	“
Sterol close to pentanorlanostane (**245**)	“	“	“
Asperphenin A (**246**)	*Aspergillus* sp.	“	[122]
Pyrenosetin A **(247**)	*Pyrenochaetopsis* sp.	“	[123]
Pyrenosetin B **(248**)	“	“	“
Pyrenosetin C **(249**)	“	“	“
Pyrenosetin D **(250**)	“	“	[124]
Radicinin (**251**)	*Cochliobolus geniculatus*	Antibiotic	[125]
Cyclo-(l-Pro-d-Pro-l-Tyr-l-Tyr) (**252**)	*Actinoalloteichus cyanogriseus*	Phytotoxic, antimicrobial	[126]
2-Hydroxyethyl-3-methyl-1,4-naphthoquinone (**253**)	“	Not reported	“
Globosuxanthone F (**254**)	*Pleosporales* sp.	“	[127]
20-Hydroxy *bis*-dechlorogeodin (**255**)	“	“	“
Dichocetide D (**256**)	*Dichotomomyces cejpii*	“	[128]
1,3,6-Trihydroxy-7-(dihydroxypropyl)-anthraquinone (**257**)	*Thermomyces lanuginosus*	“	[129]
6-(3-Hydroxybutan-2'-yl) -3,5-dimethyltetrahydro-2*H*-pyran-2-one (**258**)	“	“	“
Fusarine (**259**)	*Fusarium* sp.	“	[130]
Fusarinate (**260**)e	***“***	*“*	***“***
Penstyrylpyrone (**261**)	*Sporothrix* sp.	“	[131, 132]
Sulochrin (**262**)	“	“	“
Camptothecin (**265**)	*Penicillium chrysogenum*	“	[133]
Nigrosporin B (**266**)	*Nigrospora oryzae*	“	[134]
Chrysomutanin (**267**)	*Penicillium chrysogenum*	“	[135]
3-Acetylchrodrimanin F (**268**)	“	“	“
3-Acetoxypentacecilide A (**269**)	“	“	“
Unguidepside C (**270**)	*Aspergillus unguis*	Antibiotic	[136]
Aspersidone B (**271**)	“	“	**“**
Agonodepside C (**272**)	“	“	**“**
Preussin (**273**)	*Aspergillus candidus*	“	[137–139]
*Epi*-aszonalenin A (**274**)	*Aspergillus terreus*	Antiangiogenic	[140]
Arthpyrone M (**275**)	*Arthrinium arundinis*	Not reported	[141]
Arthpyrone N (**276**)	“	“	“
Arthpyrone O (**277**)	“	“	“

“: identical data to the entry immediately above

Source of novel compounds has expanded even to the marine environment [57, 58]. In fact, marine-derived fungi are associated to micorganisms as corals, sea cucumber, snails, sponges, algae and mangrove plants can be isolated from water and sediments [59–61]. Thus the present section describes essentially the metabolites with anticancer activity produced by marine fungi derived from different organisms or sediments. As for the previous sections the fungal marine metabolites with anticancer activity were chronologically reported.

**Fig. 5:**
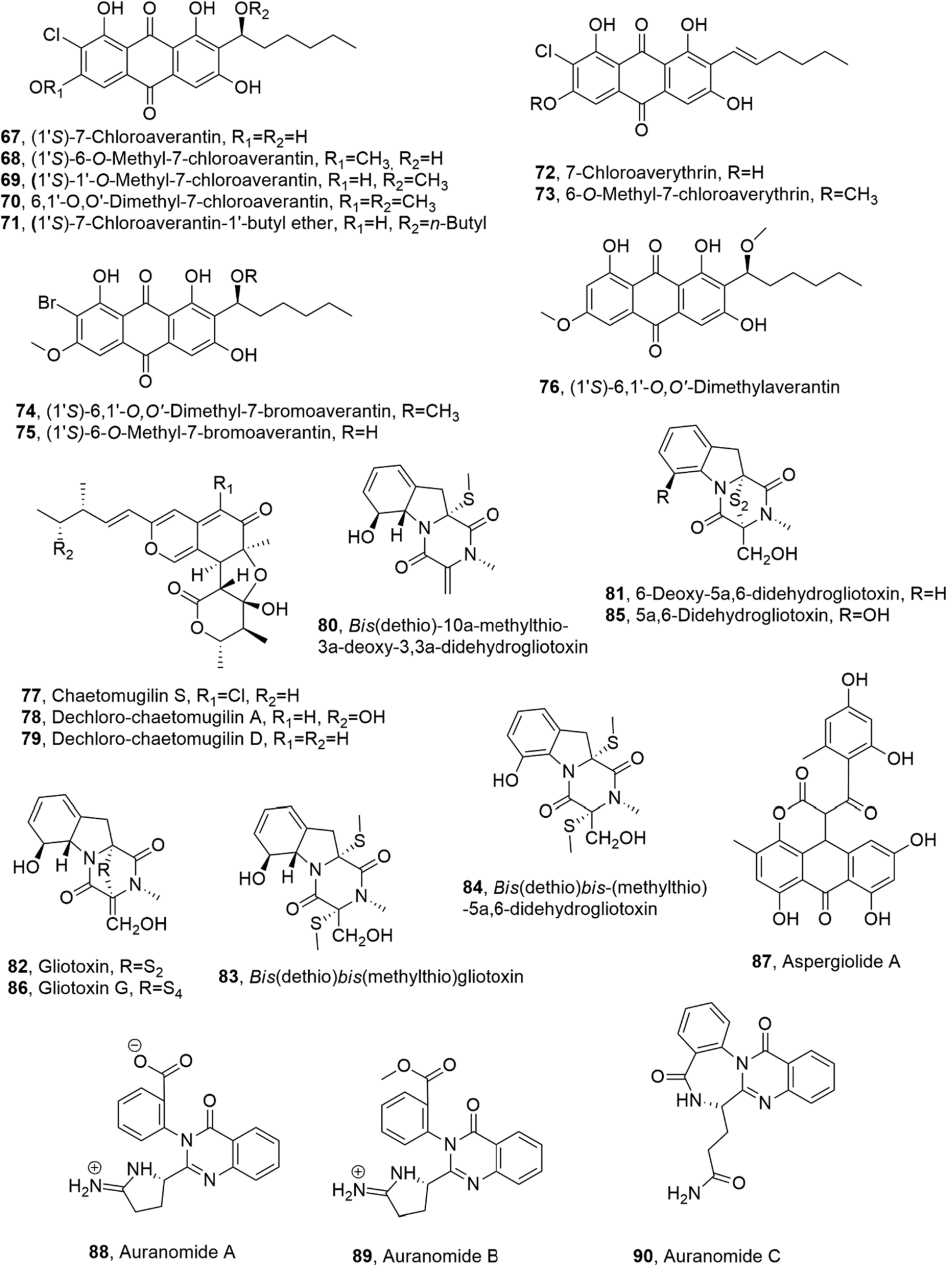
Metabolites isolated from marine fungi

Seven new chlorinated anthraquinones close to averantin as (1′*S*)-, (1′*S*)-6-*O*-methyl-, (1′*S*)-1′-*O*-methyl-, (1′*S*)-6,1′-*O,O'*-dimethyl-7-chloroaverantin, (1′*S*)-7-chloroaverantin-1′-butyl ether, 7-chloroaverythrin and 6-*O*-methyl-7-chloroaverythrin (**67**–**73**, Fig. 5) averantin, 1′-*O*-methylaverantin, 6-*O*-methylaverantin, averantin-1′-butyl ether and averythrin were isolated from the marine-derived fungus *Aspergillu*s sp. SCSIO F063 obtained from Chinese marine sediment. Two new brominated anthraquinones and non halogenated anthraquinones, namely (1′*S*)-6,1′-*O,O'*-dimethyl- and (1′*S*)-6-*O*-methyl-7-bromoaverantin and (1′*S*)-6,1′-*O,O'*-dimethylaverantin (**74**–**76**, Fig. 5) were obtained from the fungal mycelia when sodium bromide was added to the culture medium. All the compounds were assayed for cytotoxic activity and 6-*O*-methyl-7-chloroaverantin (**73**) exhibited the strongest cytotoxicity against the tumor cell lines SF-268, MCF-7, and NCI-H460 (IC_50_ values of 7.11, 6.64, and 7.42 μM, respectively). The compounds were also tested for their antibacterial activities against three Gram positive bacteria as *S. aureus* ATCC 29213, *Bacillus thuringiensis* ATCC 39765 and *Bacillus subtilis* ATCC 6633 but up to 10 μg/6 mm paper disk no toxicity was observed [62].

*Chaetomium globosum*, obtained from the marine fish *Mugil cephalus* collected in Japan, produced chaetomugilin S, dechlorochaetomugilin A and dechlorochaetomugilin D (**77**–**79**, Fig. 5). The metabolites **77**–**79** were tested for their cytotoxicity against the murine P388 leukemia cell line, the human HL-60 leukemia cell line, the murine L1210 leukemia cell line and the human KB epidermoid carcinoma cell line. Chaetomugilin S (**77**) exhibited moderate cytotoxic activity against all the cancer cell lines [63].

Bis(dethio)-10a-methylthio-3a-deoxy3,3a-didehydrogliotoxin and 6-deoxy-5a,6-didehydrogliotoxin (**80** and **81**, Fig. 5), close related to gliotoxin were isolated together with gliotoxin and gliotoxin G (**82** and **86**, Fig. 5) and their other four analogues, such as bis(dethio)bis(methylthio)gliotoxin, bis(dethio)bis-(methylthio)-5a,6-didehydrogliotoxin, 5a,6-didehydrogliotoxin, (**83**–**85**, Fig. 5), from *Penicillium* sp. strain JMF034, obtained from deep sea sediments of Suruga Bay, Japan. All the compounds were tested for their cytotoxicity against P388 murine leukemia cells. Gliotoxin and gliotoxin G (**82** and **86**) showed the strongest activity, whereas compounds **81** and **83**–**85** also had a significant activity while compound **80** exhibited only marginal activity. All the metabolites were also evaluated for their inhibitory activity against HMT G9a and HMT Set7/9 (lysine-specific histone methyltransferase for lysine 4 in histone H3) and compounds with a disulfide or tetrasulfide bond (**82**, **85**, and **86**) possessed a high inhibitory activity. Compound **81**, which also has a disulfide bond, had a weaker activity suggesting that the hydroxy group at C-6 interfers with the G9a inhibitory activity. Any compounds inhibited HMT Set7/9 at 100 μM [64].

Aspergiolide A (**87**, Fig. 5), a polyketide, was isolated from *Aspergillus glaucus* HB 1–19, a marine-derived fungus [62]. Compound **87** showed selective cytotoxic activity against A-549, HL-60, BEL-7402, and P388 cell lines with IC_50_ of 0.13, 0.28, 7.5, and 35.0 μM, respectively [65].

Auranomides A and B and C (**88**–**90**, Fig. 5), two alkaloids and a quinazolin-4-one substituted with a pyrrolidin-2-iminium moiety, were isolated together with auranthine and aurantiomides C from *Penicillium aurantiogriseum*, which is a marine-derived fungus, obtained from marine mud of the Bohai Sea, China. Compounds **88**–**90** were tested for their anticancer activity against several cancer cell lines and showed only a moderate cytotoxicity against human tumor cells. Auranomides B (**89**) exhibited the stronger activity against HEPG2 cells (IC_50_ value of 0.097 μmol/mL) [67].

**Fig. 6:**
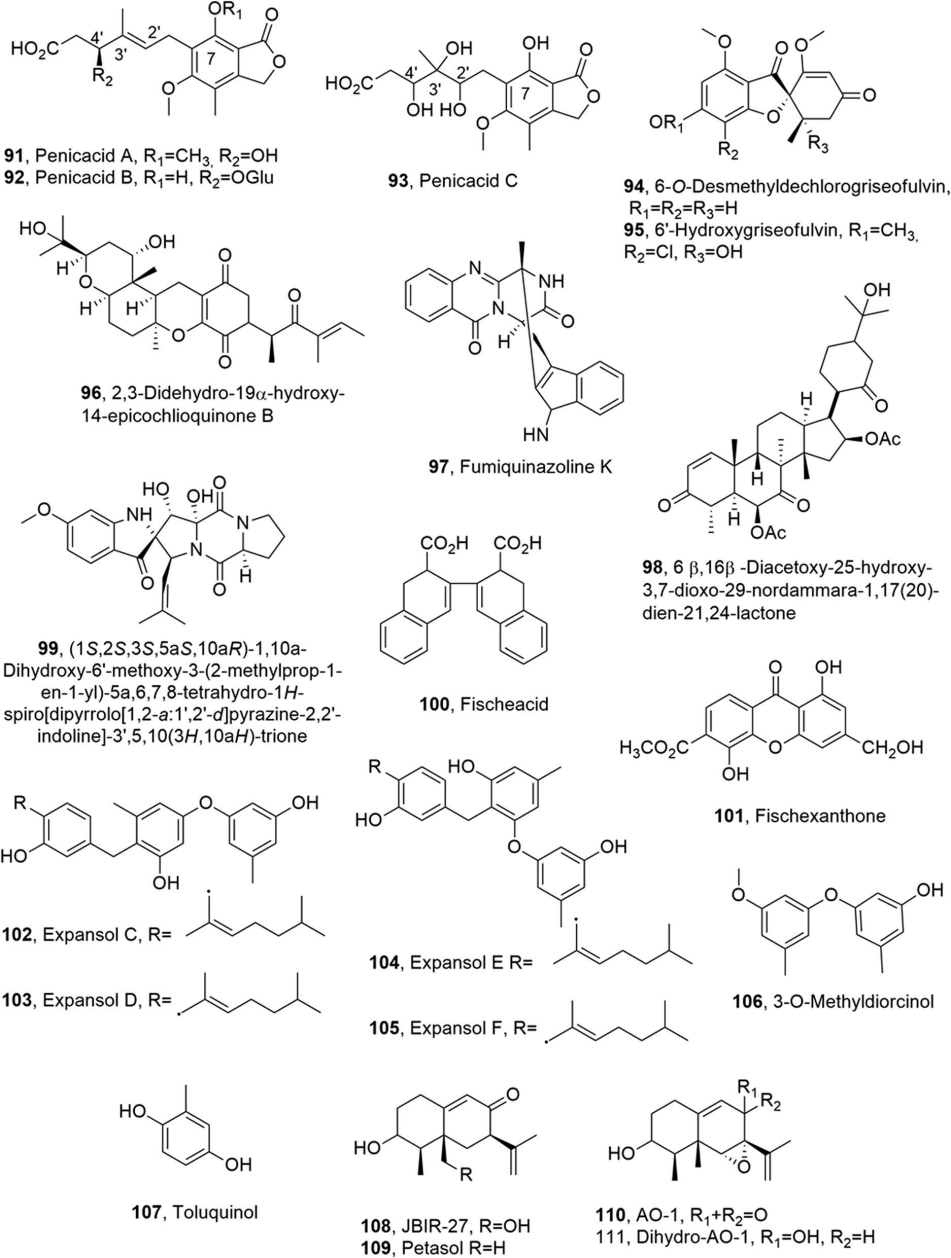
Metabolites isolated from marine fungi

Penicacids A-C (**91**–**93**, Fig. 6), three phenolic acid derivatives, were isolated together with mycophenolic acid and its 4'-hydroxy-derivative from the fungus *Penicillium* sp. SOF07, which was obtained from a Chinese marine sediment [68]. All the compounds were tested in the inhibition of IMPDH (type II) activity, which is an essential rate-limiting enzyme in the purine metabolic pathway [69]. IMPDH affects the size of the guanine nucleotide pool which, in turn, controls many physiological processes including replication, transcription, signaling and glycosylation [70] and thus it is an important drug target for immunosuppressive, antiviral and cancer chemotherapy [71]. The results obtained showed that compounds **91**–**93** and the two analogues inhibited IMPDH with IC_50_ values of 28.86, 6.43, 73.24, 0.63, and 1.79 μM, respectively. These results prompted to use the same compounds to assay their splenocyte T lymphocyte proliferation. The immunosuppresive activities of all the other compounds, at the cellular level, paralleled their IMPDH inhibitory activity, with the exception of glycosylated acid **92**. Thus SAR results from both bioassays showed that the HO-C(7), the C-2ʹ/C-3ʹ olefin, and the absence of the HO-C-4ʹ are important structural features in the immunosuppresive activities exhibited by this class of acid compounds at both the enzymatic and cellular levels [68].

6-*O*-Desmethyldechlorogriseofulvin and 6ʹ-hydroxygriseofulvin (**94** and **95**, Fig. 6) were isolated from a solid rice culture of *Nigrospora* sp. MA75, which is an endophytic fungus collected from Chinese marine semi-mangrove plant *Pongamia pinnata*. When the fungus was grown in liquid culture containing NaCl produced some other already known metabolites such as dechlorogriseofulvin and griseofulvin, the main metabolites, tetrahydrobostrycin, 4-deoxytetrahydrobostrycin, 3,8-dihydroxy-6-methoxy-1-methylxanthone, 3,6,8-trihydroxy-1-methylxanthone and griseophenone C. When NaI was added to the same liquid conditions, the fungus produced 2,3-didehydro-19a-hydroxy-14-epicochlioquinone B (**96**, Fig. 6). [72]. All the compounds were tested for their cytotoxicity and compound **96** showed activity against MCF-7, SW1990, and SMMC7721 cell lines (IC_50_ values of 4, 5, and 7 μg/ml, respectively), and moderate activity against HepG2, NCI-H460, and DU145 cell lines (IC_50_ values of 20, 11, and 17 μg/ml, respectively). It showed the stronger activity toward SW1990 cell line [72]. All the compounds were also assayed against the bacteria *S. aureus* (MRSA), *E. coli*, *Pseudomonas aeruginosa*, *Pseudomnas fluorescens* and *Staphylococcus epidermidis*, and the fungi, *C. albicans*, *Valsa mali* and *Stemphylium solani*. Compound **96** showed a significant activity against all the tested bacteria; MIC values measured were 8, 4, 4, 0.5, and 0.5 μg/ml, for MRSA, *E. coli*, *P. aeruginosa*, *P. fluorescens*, and *S. epidermidis*,respectively. Griseophenone C strongly inhibited MRSA, *E. coli*, *P. aeruginosa*, and *P. fluorescens* (MIC values of 0.5, 2, 0.5, and 0.5 μg/ml, respectively). Tetrahydrobostrycin exhibited significant activity against *MRSA* and *E. coli* (MIC values of 2 and 0.5 μg/ml, respectively), while its analog, 4-deoxytetrahydrobostrycin, showed activity only against *E. coli* with a MIC value of 4 μg/ml [72]. This results suggested that the presence of the HO-C(4) might be an important feature for the activity against MRSA. Furthermore, griseofulvin, only showed moderate activity against *V. mali* and *S. solani* (MIC values of 16 μg/ mL), while the other derivatives exhibit a weaker or no antifungal activities. The activity of griseofulvin could be due to its planar structure and spatial configuration [72]. In fact, griseofulvin is an antibiotic fungicide and is now used for the treatment of human mycotic diseases, in veterinary and plant system [73].

A fumiquinazoline K, 6β,16β-Diacetoxy-25-hydroxy-3,7-dioxo-29-nordammara-17(20)-dien-21,24-lactone (**97** and **98**, Fig. 6), an alkaloid and a nordammarane triterpenoid, were isolated together with three known diketopiperazines including spirotriprostatin A, 6-methoxyspirotriprostatin B and tryptoquivaline from *Aspergillus fumigatus* KMM 4631 associated with the soft coral *Sinularia* sp. To a third diketopiperazione was not assigned a common name but it is (1*S*,2*S*,3*S*,5a*S*,10a*R*)-1,10a-dihydroxy-6ʹ-methoxy-3-(2-methylprop-1-en-1-yl)-5a,6,7,8-tetrahydro-1*H*-spiro[dipyrrolo[1,2-a:1ʹ,2ʹ-d]pyrazine-2,2ʹ-indoline]-3ʹ,5,10(3*H*,10a*H*)-trione (**99**, Fig. 6) [74].

The marine material was collected in Kunachir island, Kuril islands, Russia. Compounds **99**, spirotriprostatin A and 6-methoxyspirotriprostatin B showed weak cytotoxic activity against cytoplasm non-specific esterase in Ehrlich carcinoma cells. Compound **99** also caused early apoptosis of the same cells using not toxic concentration range [74].

Fischeacid and fischexanthone (**100** and **101**, Fig. 6) were isolated from the culture of a marine-derived fungus *Neosartorya fischeri* strain 1008F1, together with sydowinin A, sydowinin B AGI-B4, chrysophanol, emodin, 5ʹ-deoxy-5ʹ-methylamino-adenosine, adenosine and 3,4-dihydroxybenzoic acid. All compounds were tested for their cytotoxic and antiphytoviral activity under the concentration of 200 μg/mL. Among all, AGI-B4 showed a potent inhibition of human gastric cancer cell line SGC-7901 (IC50 0.29 ± 0.005 mmol/L) and hepatic cancer cells BEL-7404 (IC50 0.31 ± 0.004 mmol/L) proliferation. The same compound and 3,4-dihydroxybenzoic acid, the main fungal metabolites, showed antiphytoviral activity with effective inhibition of the replication of TMV (Tobacco Mosaic Virus) (IC_50_ values of 0.26 ± 0.006 and 0.63 ± 0.008 mmol/L, respectively) [75].

Expansols C-F and 3-*O*-methyldiorcinol (**102**–**105** and **106**, Fig. 6), which are polyphenols containing both phenolic bisabolane and diphenyl ether moieties, and one a diphenyl ether derivative, were isolated from *Penicillium expansum* 091006. This fungus is an endogenous microrganism of the mangrove plant *Excoecaria agallocha* (Euphorbiaceae). Expansols A and B, diorcinol, and *S*-(+)-sydonic acid, (+)-(7*S*)-7-*O*-methylsydonic acid, butyrolactone I and V, WIN 64 821, 3,7-dihydroxy-1,9-dimethyldibenzofuran, orcinol, 2,4-dimethoxyphenol and 4-hydroxybenzoic acid were isolated from the same fungal culture filtrates. Among all the compounds assayed for the cytotoxic activity, expansols C and E (**102** and **104**) showed a weak cytotoxicity against the HL-60 cell lines (IC_50_ values of 18.2 and 20.8 µM, respectively). These results suggested that diphenyl ether substituted phenolic bisabolanes with a Δ^7^ double bond in the side chain are slightly lesser cytotoxic to HL-60 cell lines than derivatives having the OH or the OCH_3_ at C-7 [76].

Toluquinol (**107**, Fig. 6), a methylhydroquinone was isolated *Penicillium* sp. HL-85-ALS5- R004. Toluquinol, tested in the micromolar range, strongly inhibited activated endothelial cells and thus the growth of endothelial and tumor cells in culture. These effect seemed due to the apoptosis induction as the endothelial cell death is mediated via apoptosis after a cell cycle block and caspase activation. Furthermore, these results showed that toluquinol has antiangiogenic effects in vitro and in vivo and this activity is partly due to the suppression of the VEGF and FGF-induced Akt activation of endothelial cells [77].

JBIR-27, petasol, sporogen AO-1, and dihydro-AO-1 (**108**–**111**, Fig. 6), four sesquiterpenes, were isolated from *Penicillium citrinum* obtained from a Chinese marine coral of the Zoantharia order. AO-1 (**110**) showed antiyeast activity against *C. albicans* (MIC 4.0 mM) while it and its dihydro AO-1 (**111**) showed toxicity against Ehrlich carcinoma cells (ED_50_ of 0.9 and 0.4 mM, respectively). JBIR-27 (**108**) and petasol (**109**) were not toxic [78].

**Fig. 7:**
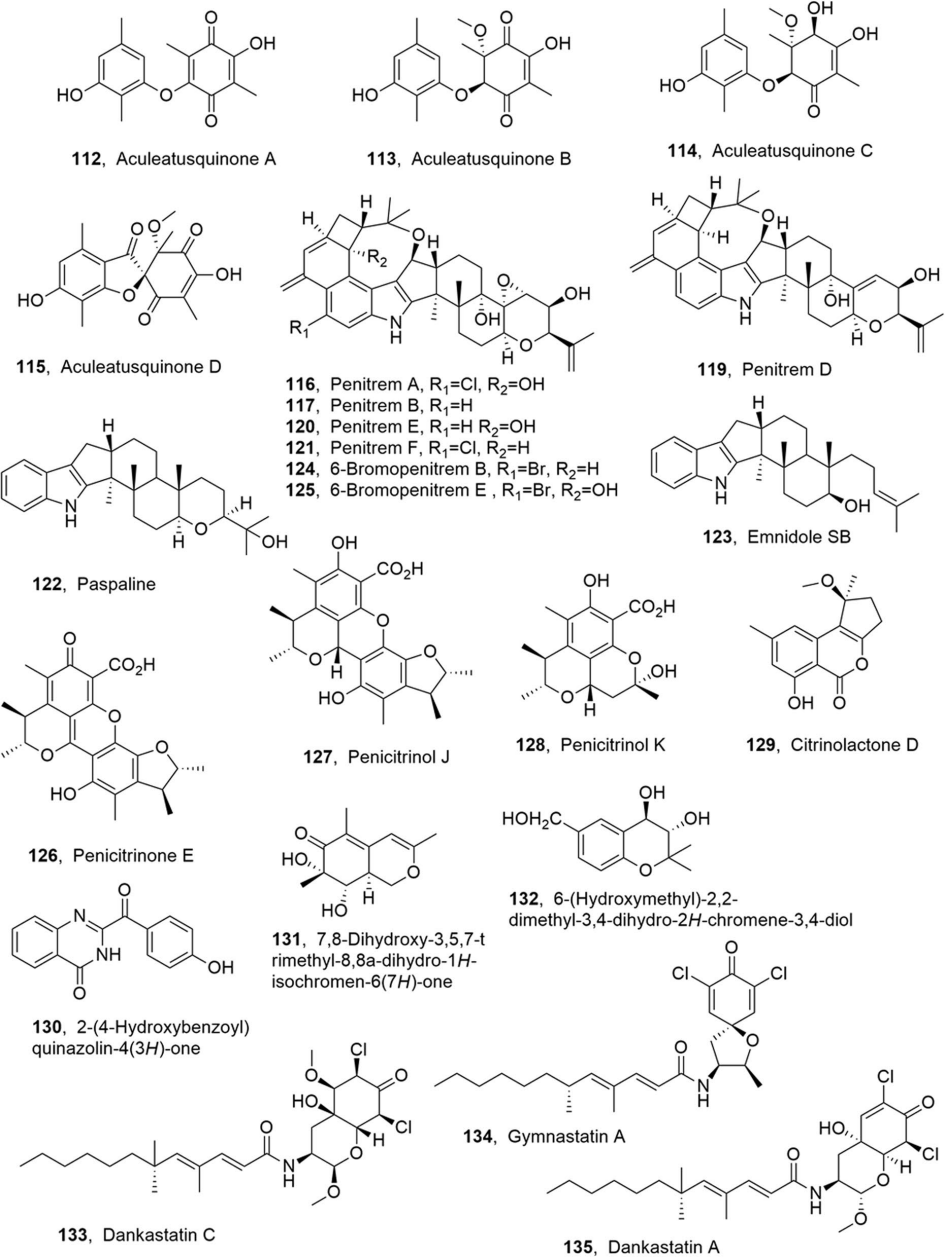
Metabolites isolated from marine fungi

Aculeatusquinones A-D (**112**–**115**, Fig. 7) were isolated from *Aspergillus aculeatus*, a marine-derived fungus, together wih 5a*S*,6*S*,7S)-3,7-dihydroxy-6-methoxy-1,4,6,9tetramethyl-6,7-dihydro-*5aH*-dibenzo[b,e][1,4]dioxepine-8,11-dione, 3,8-dihydroxy-1,4,6,9-tetramethyldibenzo[b,e][1,4]dioxepin-11-one,  4-*O*-demethylbarbatic acid, atraric acid and 2,5-dimethyl-1,3-benzenediol. Aculeatusquinones A and D (**112** and **115**) showed cytotoxic effects on the HL-60, K562, and A-549 cell lines, (IC_50_ values ranging from 5.4 to 76.1 µM) [79].

Penitrems A, B, D, E and F, paspaline and emnidole SB (**116**–**123**, Fig. 7) were isolated from *Penicillium commune* collected from the Kuwaiti coast. When KBr was added to the culture medium growth also 6-bromopenitrem B and 6-bromopenitrem E (**124** and **125**, Fig. 7) were obtained. All the metabolites showed good antiproliferative, antimigratory and antiinvasive activity against human breast cancer cells. Penitrem B (**117**) also exhibited a good toxicity in the NCI-60 DTP human tumor cell line screen. The BK channel inhibitory potential of paspaline, emnidole SB and 6-bromopenitrem B (**122**–**124**), was tested using the nematode *Caenorhabditis elegans* as an in vivo model. The same test was used to evaluated the toxicity of compounds **116**–**124**.The BK channel inhibition in *C. elegans* appeared associated with an abnormal behavior of worm locomotion in terms of increased reversals, i.e., the number of times a worm stops and reverses its direction, which can be easily assessed and quantified. Among all penitrems, penitrem A (**116**), was the most potent tremorgen and caused a reversal pattern comparable to that of the knockout strain. 6-Bromopenitrem E (**125**) had the same inhibitory activity indicating no specific halogenation preference for the activity, while emindole SB (**123**), was not active. Considering their antiproliferative activity against the breast cancer MCF-7 a pharmacophore model was produced to justify some structural relationships of **116**–**124**. Thus paspaline (**122**) and emindole SB (**123**), which are the less complex biosynthetic precursors, were identified as potential tools suitable for future studies [80].

Penicitrinone E and penicitrinol J (**126** and **127**, Fig. 7), two citrinin dimers, and two monomer derivatives penicitrinol K and citrinolactone D (**128** and **129**, Fig. 7) were isolated together penicitrinone A, penicitrinone B, citrinolactone B, citrinin, 2,3,4-trimethyl-5,7-dihydroxy-2,3-dihydrobenzofuran and phenol A from *Penicillim* sp., colleceted from the Taiwan Strait, China. All the compounds were assayed against the HeLa and HepG-2 cell lines and had no remarkable cytotoxic activity at 10 μg/mL. The antibiotic and fungicide activity of compounds **126**–**129** was tested against *S. aureus*, *E. coli*, *C. albicans* and *Aspergillus niger* and only penicitrinol J and penicitrinol K (**127** and **128**) exhibited weak antimicrobial activity against *S. aureus* CMCC26003 [81].

2-(4-Hydroxybenzoyl)quinazolin-4(3*H*)-one (**130**, Fig. 7) was isolated together with 2-(4-hydroxybenzyl)quinazolin-4(3*H*)-one, rubinaphthin A, citreorosein and methyl 4-hydroxyphenylacetate from *Penicillium oxalicum* 0312F1. The compounds were tested for their anti-phytoviral activity, at 200 μg/mL, and 2-(4-hydroxybenzyl) quinazolin-4(3*H*)-one and methyl 4-hydroxyphenylacetate showed a strong inhibition of the replication of TMV (IC_50_ values 100.80 and 137.78 μg/mL, respectively), while 2-(4-hydroxybenzoyl) quinazolin-4(3*H*)-one and rubinaphthin A exhibited moderate inhibitory activity. Among all the compounds tested at 200 μg/mL only 2-(4-hydroxybenzoyl)quinazolin-4(3*H*)-one exhibited moderate inhibitory activity of human gastric cancer cell SGC-7901 proliferation [82].

7,8-Dihydroxy-3,5,7-trimethyl-8,8a-dihydro-1*H*-isochromen-6(7*H*)-one and 6-(hydroxylmethyl)-2,2-dimethyl-3,4-dihydro-2*H*-chromene-3,4-diol (**131** and **132**, Fig. 7), two polyketides, were isolated together with [12]-cytochalasin from culture filtrates *Eutypella scoparia* FS26, a marine sediment-derived fungus obtained from the South China Sea. The three compounds were tested for their cytotoxic activity against SF-268, MCF-7 and NCI-H460, three human tumour cell lines, and [12]cytochalasin showed moderate cytoxiticity towards SF-268 and MCF-7 (IC_50_ values of 35.4 and 47.2 μM, respectively), while the two polyketides had not appreciable toxicity [83].

Dankastatin C (**133**, Fig. 7), a new polyketide tyrosine derivative, was isolated from the *Gymnascella dankaliensis*, a sponge-derived fungus, together with the steroid demethylincisterol A3, which was also previously produced by a *Homaxinella* marine sponge [84]. The compounds were assayed against the murine P388 lymphocytic leukemia cell line and dankastatin C (**133**) showed an ED_50_ value (57 ng/mL) which is similar to that of 5-fluorouracil (ED_50_ 78 ng/mL), which is the anticancer drug used as a positive control. The steroid had significant cytotoxicity (ED_50_ value of 1.0 μg/mL). The strong cytotoxic effect of the gymanastatin class of compounds probably could derived from conjugated ketones as previouslyo bserved in the close gymnastatin A [85] and dankastatin A [86] (**134** and **135**, Fig. 7), previously isolated from the same fungus, and showing ED_50_ values of 18 and 150 ng/mL, respectivly. Consequently, the potent cytotoxicity of compound **133** could be due to the presence of a conjugated ketone that this metabolite might produce in the bioassay system [87].

**Fig. 8:**
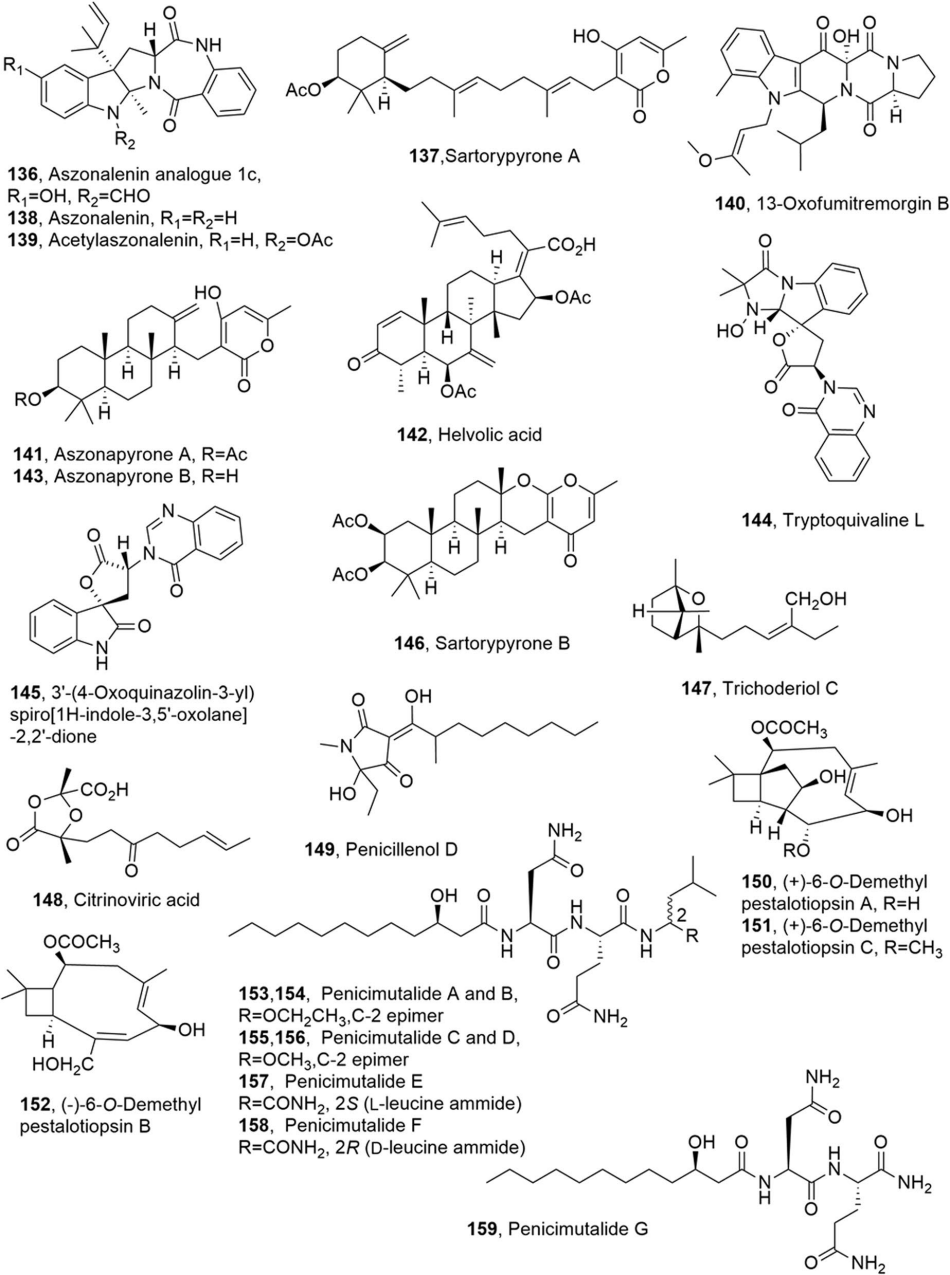
Metabolites isolated from marine fungi

Aszonalenin analogue and sartorypyrone A (**136** and **137**, Fig. 8) were isolated from the culture of the soil fungus *Neosartorya fischeri* (KUFC 6344) together with aszonalenin acetylaszon-alenin, 13-oxofumitremorgin B, aszonapyrone A and helvolic acid (**138**–**142**, Fig. 8). *Neosartorya laciniosa* (KUFC 7896), obtained from diseased coral, synthesized aszonapyrone A (**141**), aszonapyrone B, tryptoquivaline L and 3′-(4-oxoquinazolin-3-yl)spiro[1*H*-indole-3,5'-oxolane]-2,2'-dione, (**143**–**145**, Fig. 8), while *Neosartorya tsunodae* (KUFC 9213), obtained from sponge, produced sartorypyrone B (**146**, Fig. 8) and helvolic acid (**142**). Among all the metabolites isolated aszonalenin derivatives, sartorypyrone A, 13-oxofumitremorgin B, aszonapyrone A, aszonapyrone B and sartorypyrone B (**136**, **13**8, **139**, **137**, **140**, **141**, **143** and **146**), were tested against MCF-7, NCI-H460 and A375-C, brest, brest adenocarcinoma, non small cell lung cancer and melanoma cell lines, respectively [88].

Aszonapyrone A (**141**) apperared to be the most growth inhibitory compound of all the three cell lines (GI_50_ = 115.0 ± 20.0, 123.3 ± 11.5 and 68.9 ± 129 μM for MCF-7, NCI-H460 and A375-C5, respectively). Aszonapyrone B (**14**3), which differs from aszonapyrone A (**141**) for the hydrolysis of acetoxy group at C-3, was inactive also at the highest concentration tested (150μM). Sartorypyrone B (**146**) was lesser active than aszonapyrone A (**14**1), with GI_50_ 17.8 ± 7.4, 20.5 ± 2.4 and 25.0 ± 4.4 μMfor MCF-7, NCI-H460 A375-C5, respectively. Furthermore, sartorypyrone A (**137**), which include a monocyclic diterpene moiety, showed a selective inhibitory activity similar to that of sartorypyrone B (**146**) against A375-C5 cells (GI_50_ = 21.5 ± 1.9 μM), and was less active against MCF-7 and NCI-H460 (GI_50_ = 46.3 ± 7.6 and 37.3 ± 4.0 μM, respectively). Finally, the three aszonalenin derivatives **136**, **138** and **139** were no toxic against all the three cell lines at the highest concentration tested (150 μM), while13-oxofumitremorgin B (**140**) showed only weak inhibitory activity [88].

Trichoderiol C, citrinoviric acid and penicillenol D (**147**–**149**, Fig. 8) were isolated from the marine-derived fungus *Trichoderma citrinoviride*, collected from sediment of the Min River estuary in China, together with trichoderiol A, lignoren, penicillenol B1, penicillenol B2, cyclo-(Leu-Pro), cyclo-(Ile-Pro) and cyclo-(Phe-Pro). Among these compounds, **148** and **149** showed moderate cytotoxic activity against A-375 cell line, with IC_50_ values of 85.7 and 32.6 μM, respectively [89].

(+)-6-*O*-Demethylpestalotiopsin A, (+)-6-*O*-demethylpestalotiopsin C and (−)-6-*O*-demethylpestalotiopsin B (**150**–**152**, Fig. 8) were isolated from the marine-derived fungus *Ascotricha* sp. ZJ-M-5, collected on the coastal beach in Fenghua County, Zhejiang Province, China together with 1,3,6-trihydroxy-8-methylxanthone. The compounds **150**–**152** were tested for their growth inhibitory activity against HL-60 and K562 cells and metabolite **152** was not toxic (GI_50_ > 100 μM). Compounds **150** and **151** showed higher inhibition than the positive control cisplatin (GI_50_ = 13.4 ± 1.9 μM against HL-60 and 19.1 ± 2.3 mM against K562) with GI50 values of 6.9 ± 0.4 μM (HL-60) and 10.1 ± 0.9 μM (K562) for **150** and 8.5 ± 0.7 μM (HL-60) and 12.3 ± 1.1 μM (K562) for **151** [90]

Penicimutalides A-G (**153**–**159**, Fig. 8) were isolated from a mutant of a marine-derived *Penicillium purpurogenum* together with fellutamide B, fellutamide C, 1ʹ-*O*-methylaverantin, averantin. Their cytotoxic activity was tested against human cancer K562, HL-60, HeLa, BGC-823, and MCF-7 cells. The seven penicimutalides A-G (**153**–**159**) and fellutamide C weakly inhibited these cells to varying extents with inhibition rate (IR%) values as well as the five polyketides such as 1'-*O*-methylaverantin, averantin, averufin, nidurufin and sterigmatocystin against the K562 cells with showed IR% values of 11.6%, 51.9%, 37.9%, 25.5% at 100 µg/mL, and 37.5%) at 50 µg/mL. Instead, fellutamide B exhibited stronger cytotoxicity than penicimutalides A-G (**153**–**159**) and the other metabolites (IC_50_ values of 29.1, 59.9, 59.5, 77.9 and 43.3 µg/mL for K562, HL-60, HeLa, BGC-823 for MCF-7 cell lines, respectively). The positive control 5-flurouracel (5-FU) inhibited the same cell lines with the IR% values of 48.5%, 38.2%, 37.4%, 47.8% and 47.4% at 100 µg/mL [91].

**Fig. 9:**
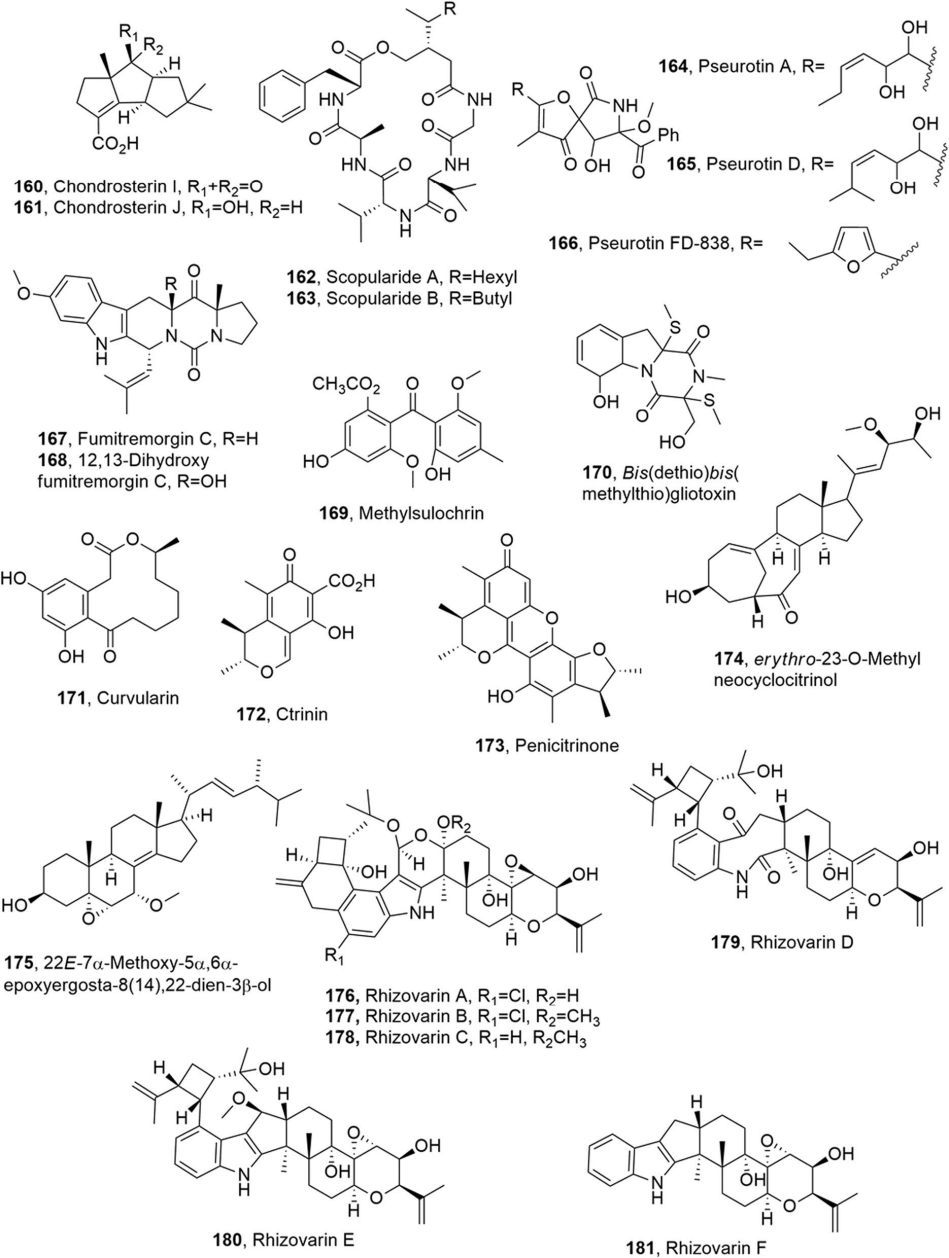
Metabolites isolated from marine fungi

Chondrosterins I and J (**160** and **161**, Fig. 9) were isolated from the marine fungus *Chondrostereum* sp., obtained from soft coral *Sarcophyton tortuosum*, collected from the Hainan Sanya National Coral Reef Reserve, China. Compounds **160** and **161** were assayed for their cytotoxicity against human nasopharyngeal cancer cell lines CNE-1 and CNE-2.Chondrosterins J (**161**) showed potent cytotoxic activity against both cell lines (IC_50_ values of 1.32 and 0.56 μM, respectively) which was stronger than that of chondrosterin A (CNE-2: 4.95 μM), hirsutanol A (CNE-1: 10.08 μM; CNE-2: 12.72 μM), and incarnal (CNE-1: 34.13 μM; CNE-2: 24.87 μM). Chondrosterin I (**160**), was not toxic (IC_50_ values > 200 μM) [92].

Scopularides A and B (**162** and **163**, Fig. 9), two cyclodepsipeptides, were isolated from *Scopulariopsis brevicaulis* LF580, which was collected from the inner tissue of the marine sponge *Tethya aurantium*. The two compounds showed specific activity against the pancreatic (Colo357, Panc89) and the colon (HT29) tumor cell lines. Thus the development of a sustainable biotechnological production process for these compounds appeared to be an important goal. A robust and reliable screening system generally applicable for the search of secondary metabolites in fungi was realized and a mutagenesis experiment was chosen as a respective application example [93].

Pseurotin A, pseurotin D and pseurotin FD-838 (**164**–**166**, Fig. 9), hetero-spirocyclic γ-lactams, and the alkaloids fumitremorgin C, 12,13-dihydroxy fumitremorgin C, methylsulochrin and bis(dethio)bis(methylthio)gliotoxin (**167**–**170**, Fig. 9) were isolated from a strain of *Aspergillus* sp. (BRF 030). The fungus was obtained from the sediments collected in the northeast coast of Brazil. All compounds were tested for their cytotoxicity against the tumour cell line HCT-116 (human colon carcinoma). Pseurotin A (**164**) showed an IC_50_ of 72 μM which did not justify the high toxicity showed by corresponding mother-fraction, while pseurotin D (**165**) had a comparable activity with IC_50_ of 85 μM. The diketopiperazines fumitremorgin C and 12,13-dihydroxy-fumitremorgin C (**167** and **16**8), appeared to be the most active compounds with an IC_50_ value of 15.17 and 4.53 μM, respectively. Pseurotin FD-838, methylsulochrin and bis(dethio)bis(methylthio)gliotoxin (**166**, **169** and **170**) showed no toxicity in the range of concentrations tested (IC_50_ > 120 μM) [94].

4-Me-6*E*,8*E*-hexadecadienoic acid was isolated from marine-derived fungus *Clonostachys rosea* collected from sediments of the river Loire estuary (France). Thes fatty acid reduced viability of MCF-7 breast cancer cells in a dose dependent manner (up to 63%) at physiological free fatty acid human plasma concentration (100 μM). Studies were also performed on its mode of action investigating the reduction of gene expression of the acetyl CoA carboxylase (ACC) and the fatty acid synthase (FAS). At 50 μM, inhibition of 50% and 35% of mRNA gene expression were observed for ACC and FAS, respectively [95].

Curvularin, citrinin, penicitrinone A, erythro-23-*O*-methylneocyclocitrinol and 22*E*-7α-methoxy-5α,6α-epoxyergosta-8(14),22-dien-3β-ol (**171**–**175**, Fig. 9) were isolated from a mutant of wild-type *Penicillium purpurogenum* G5, which was collected at the tideland of Bohai Bay around Lüjühe in Tanggu District of Tianjin, China. All the compounds were tested for their cytotoxicity against the human cancer K562, HL-60, HeLa and BGC-823 cell lines, which growth was inhibited in the ranging values (IR%) 27.5% to 88.5% at the 100 μg/mL, while the positive control docetaxol inhibited these cell lines with the IR% values of 79.9% 86.9% 78.6% and 61.5% at 100 μg/mL [96]

Rhizovarins A-F (**176**–**181**, Fig. 9) were isolated from the fungus *Mucor irregularis* (formerly known as *Rhizomucor variabilis*) collected from mangrove plant *Rhizophora stylosa*, which grows in Hainan Island, China. Some indole-diterpenes, including secopenitrem D, PC-M4, penijanthine A, paxilline, 1′-*O*-acetylpaxilline, 4b-deoxy-1′-*O*-acetylpaxilline, 3-deoxo-4b-deoxypaxilline and 3b-hydroxy4b-desoxypaxilline were obtained from the same fungus. Among all the rhizovarins, compounds **176**–**178** represent the most complex members of the reported indole-diterpenes for the presence of an unusual acetal linked to a hemiketal (**176**) or a ketal (**177** and **178**) in an unprecedented 4,6,6,8,5,6,6,6,6-fused indole-diterpene ring system. The compounds were tested for their antitumor activity against the human A-549 and HL-60 cancer cell lines. Compounds **176**, **177**, penitrems A, B and F and 3b-hydroxy4b-desoxypaxilline showed activity, while rhizovarin E (**180**) showed toxicity only against the A-549 cancer cell line. The other indole-diterpenes showed weak or no activity (IC50 > 10 μM) against these two cell lines. In this screening, all of the chlorinated compounds (**176**, **177**, penitrems A, B and F and 3b-hydroxy4b-desoxypaxilline) exhibited toxicity against both A-549 and HL-60 cancer cell lines. On the other hand, the chlorinated derivatives except **176**, showed stronger activity than their chlorine-free analogues. These results indicated that the chlorine might be an essential feature for the activity against the cell targets. Among the paxilline-type indole-diterpenes only 3b-hydroxy-4b-desoxypaxilline, in which the 13-hydroxy group is missing and the 10-keto group is replaced by 10β-hydroxy group, showed activitiy against the two cell lines. This result also suggested that the 10β-hydroxy is an essential feature to impart the activity of the paxilline type indole-diterpenes as the analogue 3-deoxo-4b-deoxypaxilline was inactive [97].

**Fig. 10:**
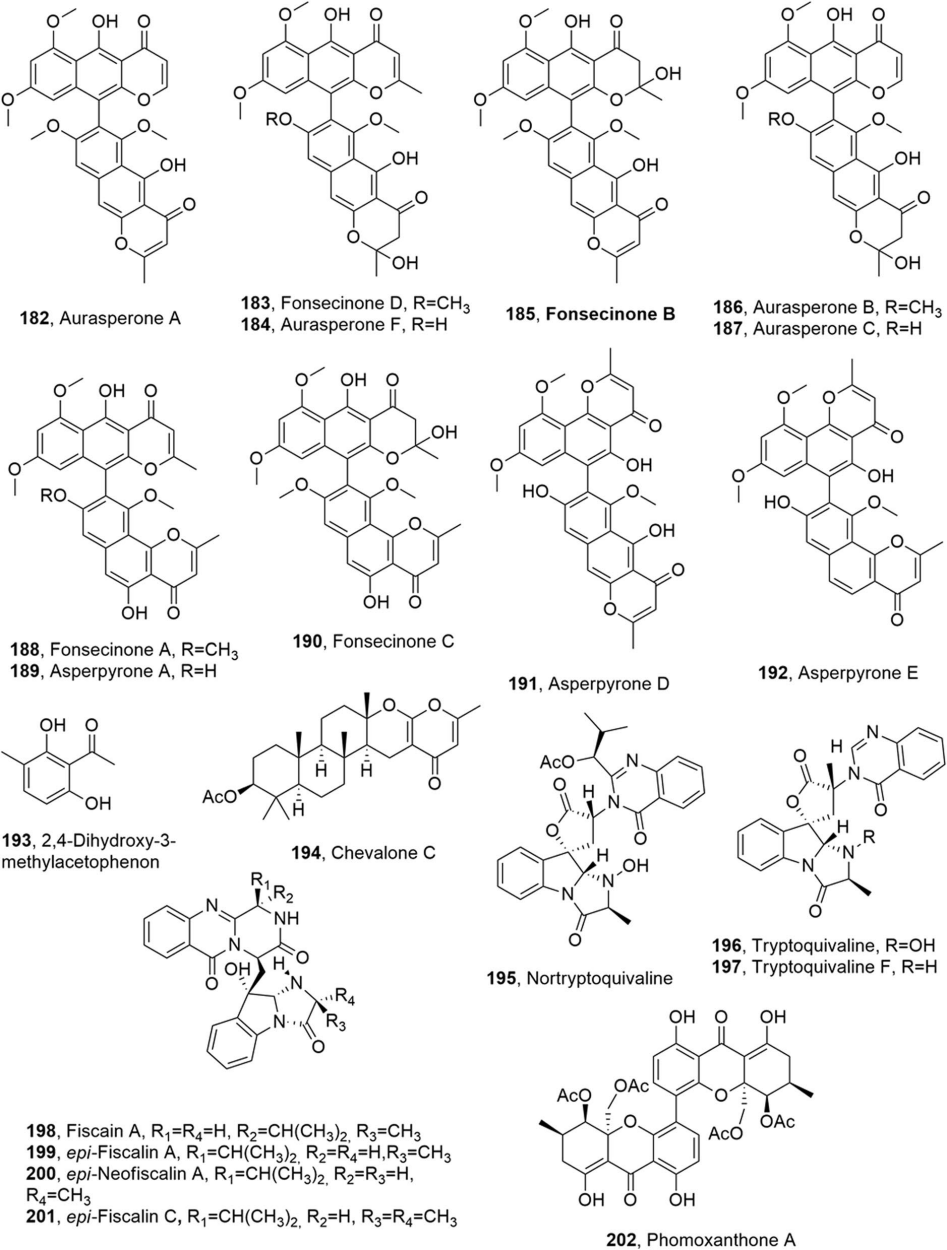
Metabolites isolated from marine fungi

Aurasperone A, fonsecinone D, aurasperone F, fonsecinone B, aurasperone B, aurasperone C, fonsecinone A, asperpyrone A, fonsecinone C, asperpyrone D, asperpyrone E (**182**–**191**, Fig. 10), which are 11-bis-naphtho-γ-pyrones (BNPs), an important subgroup of ployketides, were isolated from *Aspergillus niger* SCSIO Jcsw6F30, which was obtained from a marine alga, *Sargassum* sp collected the Yongxing Island, South China Sea. All the BPNs were assayed against 10 human cancer cell lines (K562, A549, Du145, H1975, MCF-7, Huh-7, HL7702, HL60, HeLa, and Molt-4) showing a very weak cytotoxicity (IC50 > 30 µM).

Among them, aurasperone F (**184**) exhibited the relative strongest cytotoxic activity, with the best inhibitory rates of 38.8%, 41.0%, 44.9%, 46.6%, and 49.3% against HeLa, MCF-7, Molt-4, Huh-7, and H1975, respectively, at the concentration of 30 µM. Furthermore, aurasperone, aurasperone C and asperpyrone A, (**184**, **187** and **189**) with a C-8 phenolic OH group in the structure, exhibited obvious COX-2–inhibitory activities (IC_50_ values being 11.1, 4.2, and 6.4 µM, respectively) [98].

2,4-Dihydroxy-3-methylacetophenon, chevalone C, nortryptoquivaline, tryptoquivaline H, tryptoquivaline F, fiscalin A, *epi*-fiscalin A, *epi-*neofiscalin A and *epi*-fiscalin C (**193**–**201**, Fig. 10). were isolated from *Neosartorya siamensis* (KUFA 0017), which was obtained from *Rumphella* sp., collected from the coral reef of the Similan islands, Phang Nga province, Southern Thailand. All the metabolites were tested for their anti-proliferative activity, DNA damage induction and induction of cell death on colon HCT116, liver HepG2 and melanoma A375 cancer cell lines. Compounds **194**, **195**, and **198**–**201** had IC_50_ values ranging from 24 to 153 μM on the selected cell lines. Compounds **194**, **195** and **198** induced cell death on HCT116, while compounds **195**, **198**–**200** induced significant cell death on HepG2. These results probably are not related to genotoxicity because none of the compounds induced significant DNA damage and suggested their potential (specifically *epi*-fiscalin C, **201**) as chemotherapeutic agents [99]. *N. siamensis* KUFA 0017 (NS) was combined with doxorubicin (Dox), which is one of the most successful anticancer drugs in use, and tested against six cancer cell lines. The fungal extract induced a strong enhancement of Dox's cytotoxic activity in A549 cells, causing DNA damage, cell death, and intracellular accumulation of Dox. Furthermore, 2,4-dihydroxy-3-methylacetophenone, nortryptoquivaline, chevalone C, tryptoquivaline H, fiscalin A, *epi*-fiscalin-C, *epi*-neofiscalin A, and *epi*-fiscalin A (**193**–**201**) were tested alone and combined with Dox against lung cancer cells and Dox effect increased against A459 cell line with all the compounds except with compound **193** [100].

Phomoxanthone A (**202**, Fig. 10) was isolated from the endophytic fungus *Phomopsis longicolla*, associated with marine algae *Bostrychia radicans*, which was collected in the intertidal zone of Praia Dura, Ubatuba, São Paulo State, Brazil. Compound **202** was tested for its cytotoxic activity against lymphocytes and promyelocytic leukemia HL60 cells. Its genotoxicity and mutagenicity were also assayed. Aphomoxanthone did not showed cytotoxicity, genotoxicity or mutagenicity in lymphocytes at any tested concentration (0.01 to 100.0 μg/mL). Oppositely, compound **202** was highly cytotoxic, genotoxic and mutagenic against HL60 cells, associated with a high selectivity towards HL60 compared to lymphocytes, which did not showed any damage [101].

**Fig. 11:**
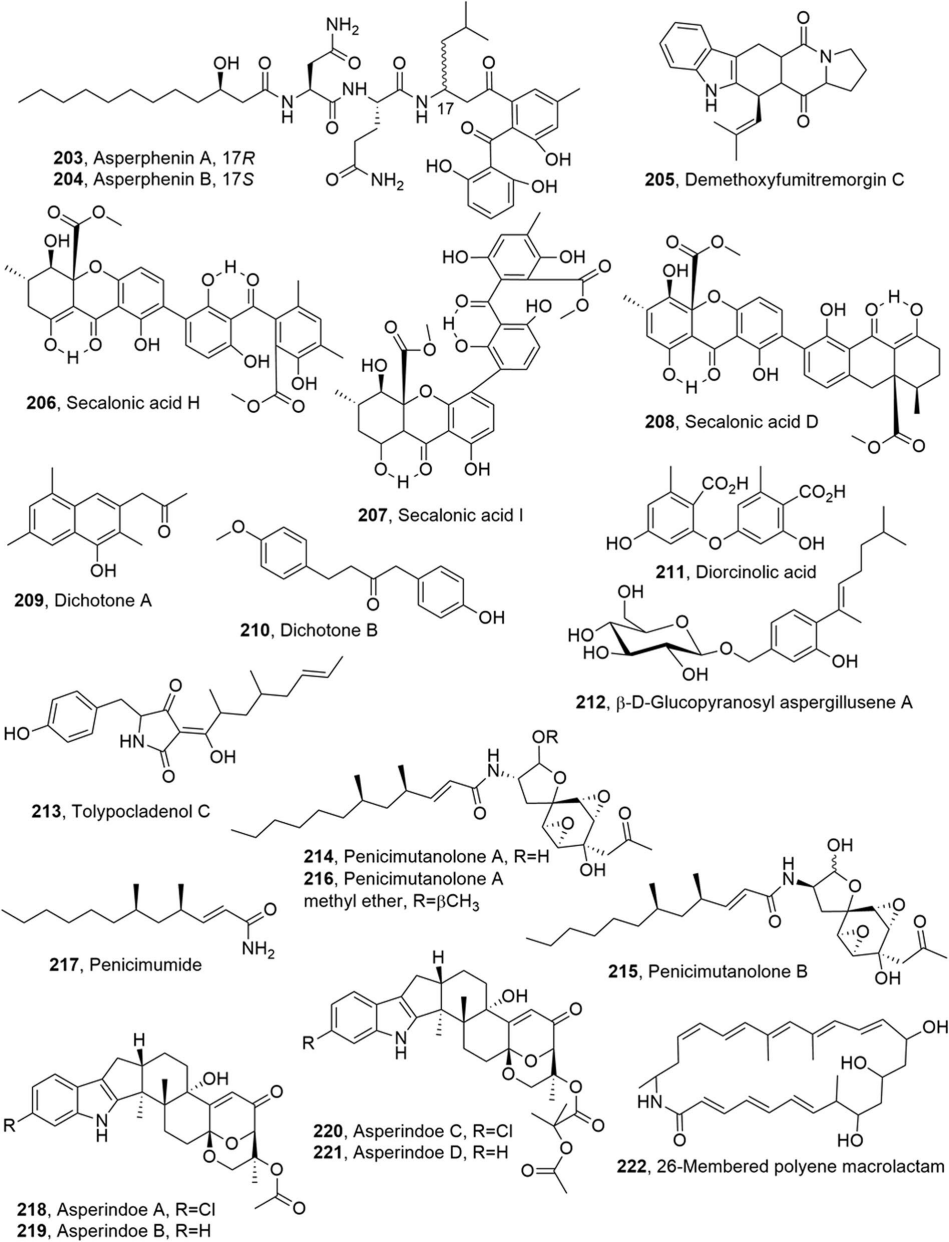
Metabolites isolated from marine fungi

Asperphenins A and B (**203** and **204**, Fig. 11), two diastereomeric lipopeptidyl benzophenones, were isolated from a marine-derived *Aspergillus* sp. fungus. These two compounds showed strong antiproliferative activity against diverse human cancer cell lines (IC_50_ values ranging from 0.8 to 9.7 μM). Among the tested cell lines, RKO colorectal carcinoma cells were the most sensitive to both compounds (IC_50_ values of 0.8 and 1.1 μM, respectively) [102].

Demethoxyfumitremorgin C (**205**, Fig. 11), was isolated from *Aspergillus fumigatus*, obtained from the surface of marine green algae, collected at Seosaeng-myeon, Ulsan, Republic of Korea. Compound **205** showed cytotoxic activity towards mouse tsFT210 cells and inhibited the cell viability of PC3 human advanced prostate cancer cells, causing apoptosis and decreased mitochondrial membrane potential. The induced apoptosis was associated with downregulation of anti-apoptotic proteins: Ras, PI3K, Akt, Bcl-xL, and Bcl-2, and upregulation of pro-apoptotic Bax. Furthermore, it activated caspase-3, -8, and -9, leading to PARP (Poli ADP ribosio polimerasi) cleavage. Caspase inhibitors blocked demethoxyfumitremorgin C (**205**) inducing apoptosis of PC3 cells. These results suggested that compound **205** inhibits the proliferation of PC3 human prostate cancer cells via mitochondrial intrinsic and extrinsic pathway, followed by downstream events leading to apoptotic cell death [103].

Secalonic acids H and I (**206** and **207**, Fig. 11), two new secalonic acid analogues, were isolated together with secalonic acid D (**208**, Fig. 11) from the marine-derived fungus *Penicillum oxalicum*, which was obtained from a sediment sample of the southeast coast of China. Compounds **206** and **207** were tested for their cytotoxicity against HCT116, KB and EC9706 cells with secalonic acids H showing stronger toxicity against three cell lines than secalonic acids I [104].

Dichotones A and B (**209** and **210**, Fig. 11) were isolated together with dichotocejpin C, bis-*N*-norgliovictin, bassiatin and (3*R*,6*R*)-bassiatin from *Dichotomomyces* sp. L-8 associated with the soft coral *Lobophytum crissum*, collected from Hainan Sanya National Coral Reef Reserve, China. Their cytotoxicity was tested and (3*R*,6*R*)-bassiatin displayed significant toxicity against the human breast cancer cell line MDA-MB-435 and the human lung cancer cell line Calu3 (IC_50_ values of 7.34 ± 0.20 and 14.54 ± 0.01 µM, respectively), while bassiatin, its diastereomer, was not toxic [105].

Diorcinolic acid and β-d-glucopyranosyl aspergillusene A (**211** and **212**, Fig. 11) were isolated together with six diphenylethers, a diketopiperazine, a chromone and a xanthone from the fungus *Aspergillus sydowii* derived from the marine sponge *Stelletta* sp. Compounds **211** and **212** showed mild cytotoxicity against KB (human nasopharyngeal carcinoma cells), HepG2 (human liver cancer cells) and HCT 116 (human colon cancer cells). They were also assayed for their antibacterial activity and their ability to suppress LPS-induced nitric oxide (NO) production. Some diphenylethers showed mild antibacterial activity against human pathogen *S. aureus* and fish pathogens *Streptococcus iniae* and *Vibrio ichthyoenteri*, and weakly suppressed NO production [106].

Tolypocladenol C (**213**, Fig. 11) was isolated together cyclosporin A, efrapeptin D, pyridoxatin, terricolin A, malettinins B and E, and tolypocladenols A1/A2 from *Tolypocladium geodes*, a fungus collected from a sponge. All compounds were tested for their anticancer activity using a selection of the NCI60 cells and all inhibited, at the highest concentration, the growth of the breast adenocarcinoma cell lines MCF-7, while those of the ovarian carcinoma cell lines OVCAR-5 were reduced to at least 50% only by four of the seven compounds. Efrapeptin D inhibited the growth of MCF‐7 and OVCAR‐5 cell lines at 7 at μM and 12 μM, respectively, while malettinins B and E exhibited an activity on every cell line used, except melanoma cell line SK‐MEL‐28, which is inhibited by malettinin B. Tolypocladenol C and tolypocladenols A1/A2 were no toxic against the majority of cancer cell lines tested, while pyridoxatin showed a good toxicity against the cell line panel in the submicromolar and low micromolar range [107].

Penicimutanolones A and B, penicimutanolone A methyl ether, and penicimumide, (**214**–**217**, Fig. 11) were isolated from a neomycin-resistant mutant of the marine-derived fungus *Penicillium purpurogenum* G59, collected at the tideland of Bohai Bay, Tanggu district of Tianjin, China. All the compounds were tested for their inhibitory activity against A549, HeLa, MCF-7, HCT116, HepG2, NCI-H1975, HL-60, K562, LS180, SW480, HT29, PC-3, BXPC-3, and PANC-1 human cell lines. As positive control was used adriamycin 1 μM and 5-fluorouracil 100 μM, which had inhibition rates that ranged from 70 to 98% and 41% and 95%, respectively. Penicimutanolones A and penicimutanolone A methyl ether (**214** and **216**) exhibited a significant inhibition of all human cancer cell lines, while compounds **214** and **217** may induce apoptosis of cancer cells essentially as consequence of the inhibition of the expression of survivin, a client protein of HSP90. Penicimutanolones showed in vivo toxicity against murine sarcoma HCT116 tumor-bearing Kunming mice, l [108].

Asperindoles A-D (**218**–**221**, Fig. 11), which are four indole-diterpene alkaloids, were isolate together with 3"-hydroxyterphenyllin from *Aspergillus* sp., a fungus associated with an unidentified colonial ascidian (Shikotan Island, Pacific Ocean). Asperindole A (**218**) showed cytotoxicity against human PC-3, LNCaP (androgen-sensitive human prostate adenocarcinoma cells), and 22Rv1 (IC_50_ values of 69.4 µM, 47.8 µM, and 4.86 µM, respectively) using docetaxel as as a positive control (IC_50_ values of 15.4 nM, 3.8 nM, and 12.7 nM, respectively). Asperindole C (**220**) was not toxic on all the three cell lines (IC_50_ > 100 µM), while asperindole A (**218**) induced apoptosis in human cancer 22Rv1 cells at low-micromolar concentrations and the cell cycle progression analysis of these cells treated with the same compound for 48 h showed a S-phase arrest (as well as a discrete G2/Mphase). Furthermore, compound **218** showed cytotoxicity towards hormone therapy-resistant PC-3 and 22Rv1 cells, as well as, hormone therapy-sensitive human prostate cancer cells inducing apoptosis at low-micromolar concentration [109].

A 26-membered polyene macrolactam (**222**, Fig. 11) was isolated together aurodox from marine-derived actinomycete strain *Micromonospora* sp. FIM05328, obtained from a soil sample collected in East China Sea. Compound **222** had toxicity towards KYSE30, KYSE180 and EC109 human tumour cell lines (IC_50_ values of 15.92 μM, 30.77 μM and 0.00020 μM, respectively). Aurodox exhibited toxicity against KYSE30, KYSE180 and EC109 cell lines (IC_50_ values of 22.52 μM, 83.76 μM and 20.56 μM, respectively). The positive control cisplatin showed against the same cell lines IC_50_ values of 1.79, 26.71 and 0.019 mM, respectively.[110].

**Fig. 12:**
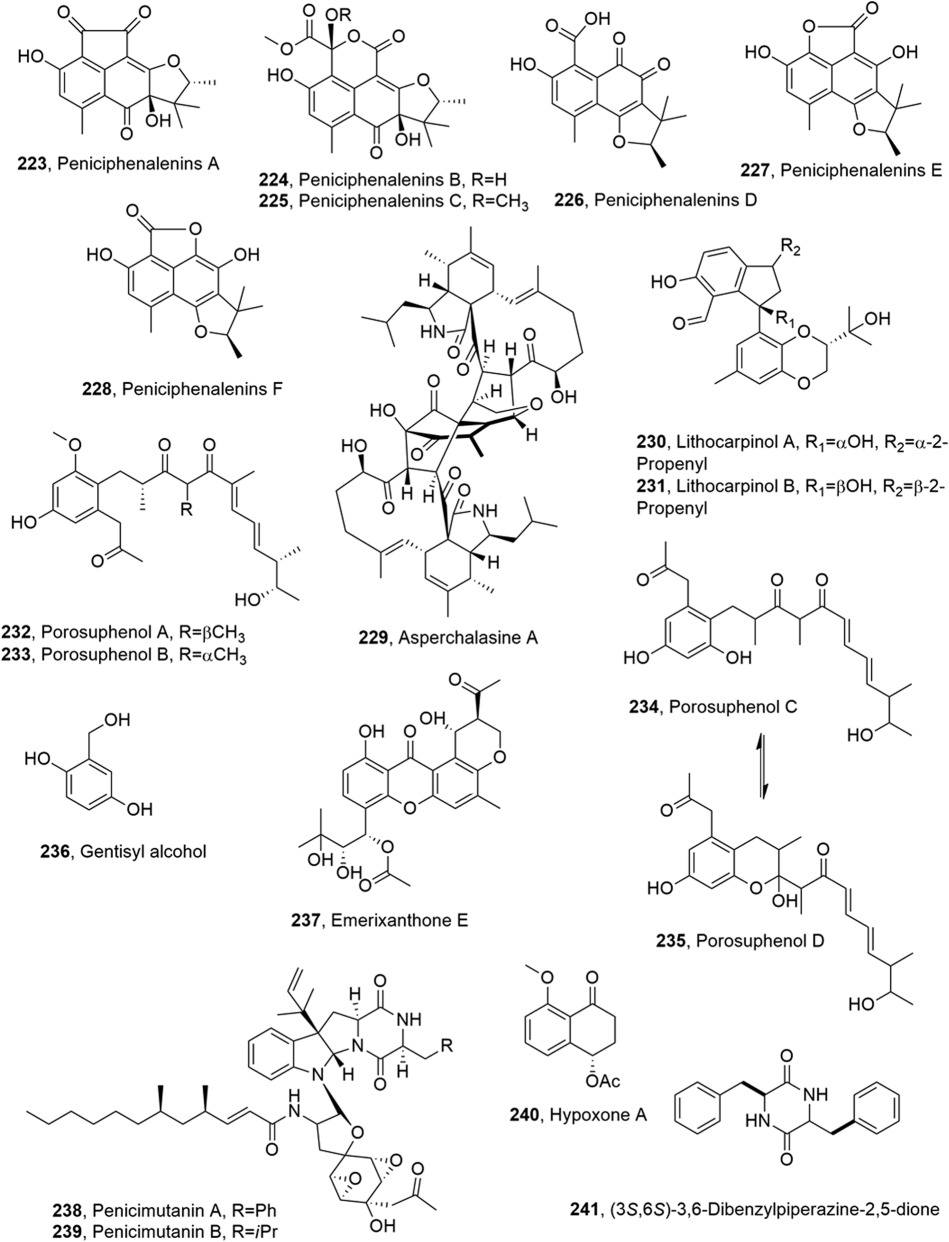
Metabolites isolated from marine fungi

Peniciphenalenins A-F (**223**–**228**, Fig. 12) were isolated together with (+)-sclerodin, (+)-scleroderolide, (+)-sclerodione, and physcion from a marine-derived *Penicillium* sp. All the compounds were tested against glioma U87MG and C6 cells proliferation using doxorubicin, a chemotherapeutic drug as a positive control. (+)-Sclerodin, (+)-scleroderolide, (+)-sclerodione, and physcion showed a weak activity against glioma cells (IC_50_ values of 44.65 and 55.99 μM, 23.24 and 37.26 μM, 60.81and 60.93 μM, and 30.22 and 34.78 μM, respectivey), while peniciphenalenins A-F (**223**–**228**) were not toxic [111].

Asperchalasine A (**229**, Fig. 12), which is a cytochalasan dimer consistsing of two cytochalasan moieties connected by an epicoccine, was isolated from the marine-derived fungus *Aspergillus flavipes*. Fungal extract and asperchalasine A (**229**) significantly inhibited cell adhesion and tube formation in human umbilical vein endothelial cells (HUVECs). Similarly, the same samples in a concentration-dependent manner, decreased the vascular endothelial growth factor (VEGF) and vascular endothelial growth factor receptor (VEGFR)-2 mRNA expression They also inhibited angiogenesis via downregulation of VEGF, p-p38, p-extracellular signal-regulated protein kinase (ERK), p-VEGFR-2, and p-Akt signaling pathways. Both fungal extract and compound **229**, using a chorioallantoic membrane assay, showed to strongly inhibited the amount of blood vessel formation in fertilized chicken eggs. These results suggested the potential antiangiogenic of asperchalasine A [112].

Lithocarpinols A and B (**230** and **231**, Fig. 12), a pair of tenellone diastereoisomers with novel fused skeleton, were isolated from the deep-sea derived fungus *Phomopsis lithocarpus* FS508. Lithocarpinols A and B were assayed for their cytotoxicity towards HepG-2, MCF-7, SF-268, and NCI-H460 cell lines using cisplatin as the positive control. Both metabolites determine the inhibition of all the tested cell lines. In particular, lithocarpinol A (**230**) exhibited a moderate inhibition effect towards HepG-2 and A549 cell lines (IC_50_ values of 9.4 and 10.9 μmol/L, respectively), while lithocarpinol B (**231**) exhibited a weak inhibitory activity (IC_50_ values in the range of μmol/L) [113].

Porosuphenols A-D (**232**–**235**, Fig. 12), four polyketides, were isolated together with sphaeropsidin A, and aspergiloid E from *Aspergillus porosus*, a marine derived fungus. Porosuphenols C and D (**234** and **235**) are in ketone-hemiketal equilibrium. All compounds were assayed for antibacterial activity against *S. aureus*, ATCC 25923 and ATCC BAA-41, and for cytotoxic activity towards carcinoma HCT-116, ATCC CCL-247. Only sphaeropsidin A showed strong toxicity despite the nearly identical structure to aspergiloid E, which only differed from the toxin for the absence of the hydroxy group at C-9. The porosuphenols A, B, and C/D (**232**–**235**) had no antibacterial and effect on cell viability assays; they also did not showed antifungal, antimalaria, antitubercular, antioxidant, and metal-chelating activity (> 50 μM) [114].

Gentisyl alcohol (**236**, Fig. 12), isolated from *Arthrinium* sp., which is a derived from marine fungus, suppressed proliferation in human ovarian cancers cells (ES2 and OV90 cells), inducing apoptosis via DNA fragmentation. Ovarian cancer cells treated with gentisyl alcohol accumulated sub-G1 cells and lost mitochondrial membrane potential with calcium dysregulation. Compound **236** up-regulated signal transduction of MAPK and PI3K/AKT pathways [115].

Emerixanthone E (**237**, Fig. 12) was isolated together with four emodin derivatives from *Emericella* sp collected from deep sea sediments in the South China Sea. Compound **237** and an emodin derivative exhibited a moderate antibiotic activity at the concentration of 50 μg/well towards *E. coli* (ATCC 29922), *Klebsiella pneumonia* (ATCC 13883), *S aureus* (ATCC 29213), *Enterococcus faecalis* (ATCC 29212), *Acinetobacter baumannii* (ATCC 19606), and *Aeromonas hydrophila* (ATCC 7966). None of the compounds displayed antifungal and antitumor activity against K-562, A-549, HL-60, Huh7, MCF-7, H-1975, U937, BGC823, Hela, and MOLT-4 cell lines [116].

Penicimutanins A and C (**238** and **239**, Fig. 12), two alkaloids, were isolated together with fructigenine B, rugulosuvine A and fructigenine A from the neomycin-resistant mutant strain 3-f-31 of the marine-derived fungus *Penicillium purpurogenum* G59. The compounds were assayed for their cytotoxicity towards K562, HL-60, HeLa, BGC-823, and MCF-7 cell lines and using 5-fluorouracil (5-FU) as a positive control. Compounds **238** and **239** exhibited stronger inhibition than the other three compounds (IC_50_ values against K562, HL-60, HeLa, BGC-823, and MCF-7 of 10.7, 6.1, 7.0, 8.3, and 7.3 μM for **238** and 11.9, 5.0, 8.6, 8.7, and 6.0 μM for **239**, respectively) Fructigenine B, rugulosuvine A and fructigenine A and 5-FU were not toxic (IC_50_ values greater than 100 μM) [117].

Hypoxone A (**240**, Fig. 12), was isolated from the marine fungus *Hypoxylon rubiginosum* FS521. 4,8-Dimethoxy-1-naphthol, 1ʹ-hydroxy-4ʹ,8,8ʹ-trimethoxy[2,2']binaphthalenyl-1,4-dione, 3,6-dimethyl-atromentin, xylarenone, regiolone, methylsclerone, 5-methylmellein, 3,5-dimethyl-8-methoxy-3,4-dihydrosiocomarin, 9-methoxynaphthalene, 1,8-dimethoxynaphthalene, 8-methoxy-1-naphthol, 7-methoxy-3-methylisoben-zofuran, 3-methyl-4-hydroxyphenyl isopropanoid, nodulisporipyrones A and B, 4-[2-[(2,2-dichloro-l-methylethenyl)oxy]ethyl]-l,2-dimethoxybenzene were also isolated from the same fungus. Compound **240** and 4,8-dimethoxy-1-naphthol, 1ʹ-hydroxy-4ʹ,8,8ʹ-trimethoxy[2,2ʹ]binaphthalenyl-1,4-dione and 3,6-dimethyl-atromentin were tested for their cytotoxicity towards SF-268, MCF-7, HepG-2, and A549 tumor cell lines, and binaphthalenyl-1,4-dione derivative showed significant activity (IC_50_ values of 1.85, 3.21, 2.53,and 5.09 μmol/L) respectively [118].

(3S,6S)-3,6-Dibenzylpiperazine-2,5-dione (**241**, Fig. 12) was isolated the marine-derived *Paecilomyces formous* 17D47-2. This compound showed citotoxiciy towards the PANC-1 human pancreatic carcinoma cells adapted to glucose-starved conditions (IC50 value of 28 µM), while up to 1000 µM no toxicity was observed against PANC-1 cells under general culture conditions. These results suggest that compound **241** may act via uncoupling of mitochondrial oxidative phosphorylation [119].

**Fig. 13:**
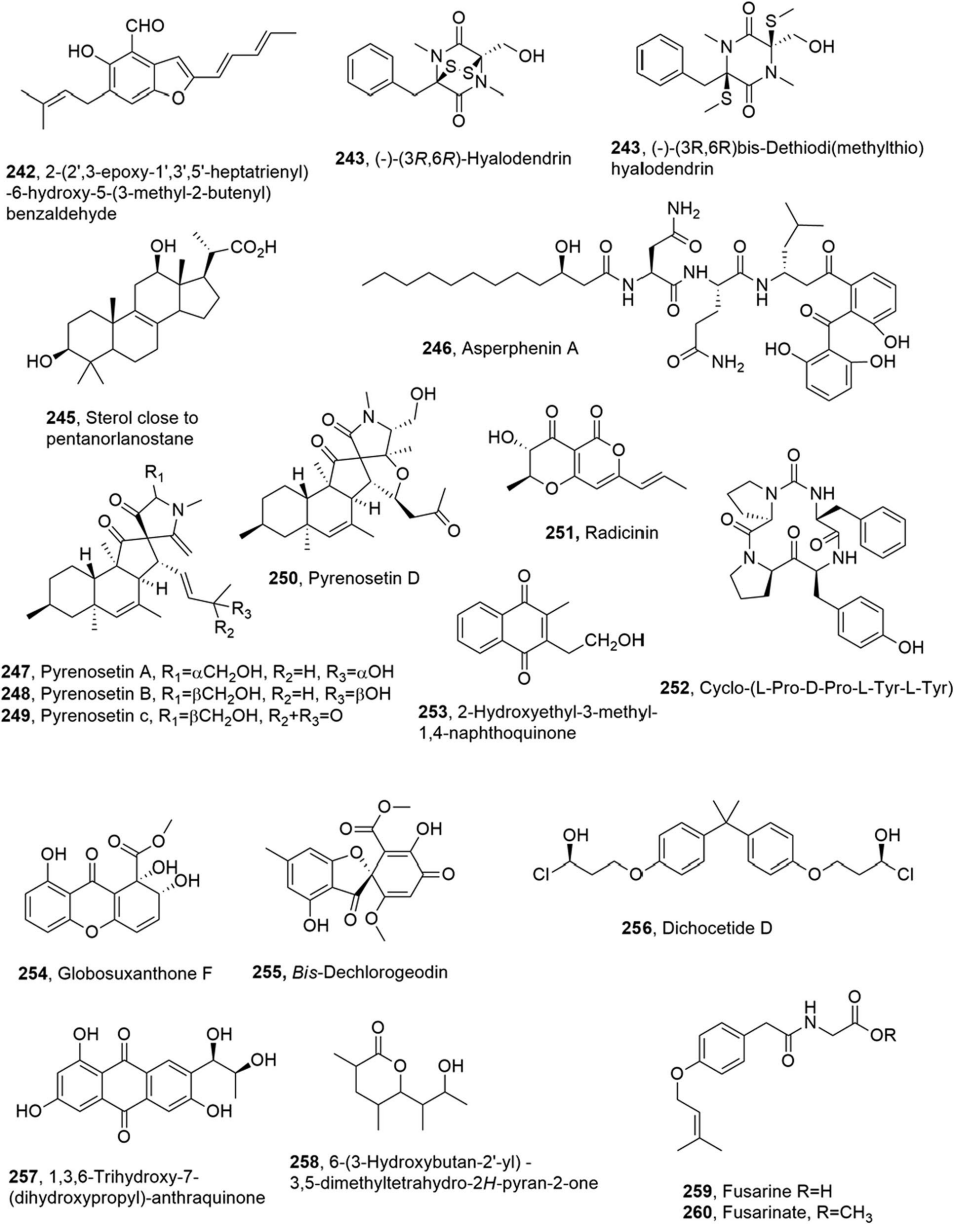
Metabolites isolated from marine fungi

2-(2ʹ,3-Epoxy-1ʹ,3ʹ,5ʹ-heptatrienyl)-6-hydroxy-5-(3-methyl-2-butenyl) benzaldehyde (**242**, Fig. 13) and physcion were isolated from a sponge-derived fungus *Aspergillus* sp. Physcion and compound **242** showed selective cytotoxicity against human pancreatic carcinoma PANC-1 (IC_50_ values of 6.0 and 1.7 µM, respectively). Compound **242** exhibited higher selective growth-inhibitory activity (505-fold higher) under glucose-deficient conditions than under general culture conditions. Further studies on their mechanism related to their anti austerity activity towards glucose-starved PANC-1 cells highlighted the ability of the two compounds to inhibit the mitochondrial electron transport chain [120].

(−)-(3*R*,6*R*)-Hyalodendrin (**243**, Fig. 13), was isolated together with (−)-(3*R*,6*R*)bis-dethiodi(methylthio)hyalodendrin, a sterol close to pentanorlanostane (**244** and **245**, Fig. 13) and glycocholic acid methyl ester from *Paradendryphiella salina* PC 362H strain, obtained from the brown alga *Pelvetia caniculata* (PC). The cytotoxicity of sterol (**245**) and glycocholic acid methyl ester was assayed towards MCF7, MCF7-Sh-WISP2 and 3T3-F442A cell lines and at a concentration as high as 50 µM they did not demonstrated toxicity. The same compounds had a weak activity against *Plasmodium falciparum* (IC_50_ values of 65 ± 4.2 and 20.5 ± 3.5 µM, respectively) Compound **244** exhibited only a modest activity against MCF7, MCF7-Sh-WISP2 and 3T3-F442A cell lines (IC_50_ values of 42, 68 and 26 µM, respectively) while compound **243**, having a disulfide bond between C-3 and C-6 instead of two methylsulfide groups, had a strong activity against the MCF7-Sh-WISP2 invasive cells (IC_50_ value of 140 nM). Interestingly, compound **243** was more active towards MCF7-Sh-WISP2 than MCF7 or 3T3-F442A. These results confirmed that members of the *epi*-dithiodioxopiperazines are intruing toxins with anticancer activities [121].

Asperphenin A (**246**, Fig. 13), a lipopeptidyl benzophenone was isolated from large-scale cultivation of marine-derived *Aspergillus* sp. Compound **246** showed strong antiproliferative activity against various cancer cells. In particular, it inhibited the growth of colon cancer cells through G2/M cell cycle arrest followed by apoptosis, triggered microtubule disassembly and induced reactive oxygen species. Compound **246** totally inhibited the tumor growth in a colon cancer xenograft model without any over toxicity and showed a combination effect with irinotecan, which is a topoisomerase I inhibitor. Studies performed using some synthetic derivatives of asperphenin A demonstrated that the aryl ketone should be a key structureal feature responsible for activity [122].

Pyrenosetins A-C (**247**–**249**, Fig. 13) were isolated together with the decalin tetramic acid phomasetin from *Pyrenochaetopsis* sp. FVE-001, an endophytic fungus collected from the brown alga *Fucus vesiculosus*. All the compounds were tested for their cytotoxic activity against the human malignant melanoma cancer cells (A-375). Compound **247** and **248** had the highest activity (IC_50_ values of 2.8 and 6.3 µM, respectivel)y. Compound **249** and phomasetin were less toxic (IC_50_ 140.3 and 37.3 µM). The toxicity of the metabolites was also assayed towards the human keratinocyte cell line HaCaT and compounds **247**, **249** and phomasetin showed IC_50_ values very similar that oberved against melanoma cells, indicating their non-selective toxicity. Compound **248** had lower toxicity towards HaCaT cells (IC_50_ 35.0 µM) with a better selective index around 5.6 compared to those of the other three fungal metabolites [123]. Pyrenosetins D (**250** Fig. 13), another decalinoyltetramic acid derivative, was isolated together with wakodecalines A and B from the same fungus *Pyrenochaetopsis* sp. FVE-00. These latter metabolites were assayed for the inhibition ability of the human malignant melanoma cell line (A-375) and the human keratinocyte cells (HaCaT). Compound **247** showed moderate anticancer activity against the A-375 cells (IC_50_ value 77.5 µM), but was toxic on the HaCaT cells (IC_50_ value of 39.3 µM), while wakodecalines A and B, even at the highest test concentration of 200 µM exhibited not toxicity [124].

Radicinin (**251**, Fig. 13) was isolated from the sponge-derived fungus *Cochliobolus geniculatus*, collected in Setan Island at Indonesia. It showed cytotoxic activity towards WiDr, T47D, and Hela cell lines (IC_50_ values of 60.68, 30.89, and 87.89 µg/mL, respectively) but was not toxic against Vero cell (IC_50_ value of 607.31 µg/mL). Radicinin exhibited higher cytotoxicity against T47D cells (IC_50_ 25.01 ppm) than doxorubicin (IC_50_ ppm) and showed antibiotic activity against MRSA with a MIC of 125 µg/disc [122].

Cyclo-(l-Pro-d-Pro-l-Tyr-l-Tyr) and 2-hydroxyethyl-3-methyl-1,4-naphthoquinone (**252** and **253**, Fig. 13) were isolated from *Actinoalloteichus cyanogriseus* a Chinese marine derived fungus. Compound **253** showed strong inhibitionof several phytopathogenic fungi including *Fusarium oxysporum* f. sp. *cucumerinum*, *Setosphaeria turcica*, and *Botrytis cinerea* and Gram-positive bacterium as *B. subtilis* and *S. aureus* DSM 346, Gram-negative bacterium as *Chromobacterium violaceum* DSM 30191, yeasts as *Rhodotorula glutinis* DSM 10134 and *C. albicans* DSM 1665, and flamentous fungi as *Mucor hiemalis* DSM 2656. Furthermore, it also had moderate cytotoxicity towards human breast cancer MDA-MB-435 cells (IC50 10.59 µM), which is weaker than that of positive control diaminedichloroplatinum (5.91 μM) [126].

Globosuxanthone F and bis-dechlorogeodin (**25**4 and **255**, Fig. 13), two polyketides, were isolated together with 3,4-dihydroglobosuxanthone A, 8-hydroxy-3-methylxanthone-1-carboxylate, crosphaeropsone C, and 4-megastigmen-3,9-dione from the sponge-derived fungus *Pleosporale*s sp. NBUF144. The compounds were assayed for their cytotoxic activity against CCRF-CEM human acute lymphatic leukemia cells. Globosuxanthone A (**254**) demonstrating toxicity towards NCI-H460, MCF-7, SF-268, PC-3, PC-3M, LNCaP, DU-145, and HCT-15 tumor cell lines, as well as, T-cell leukemia Jurkat and exhibited strong cytotoxicity in vitro against CCRF-CEM T-cell leukemia cells (IC_50_ value of 0.46 µM). Crosphaeropsone C, 3,4-dihydroglobosuxanthone A and 8-hydroxy-3-methylxanthone-1-carboxylate, when tested at at 20 µM, did not exhibited pronounced toxicity. These results highlighted that the presence of the 3,4-unsaturation and that of the hydroxy groups at C-1 and C-2 are important features to impart anticancer activity [127].

Dichocetide D (**256**, Fig. 13), containing a chlorine, was isolated together with (22*E*)-8α-epidioxyergosta-6,22-dien-3β-ol, ergosta-4,6,8(14),22-tetraene-3-one and ergosterol from the marine-derived fungus *Dichotomomyces cejpii* F31. All the compounds were tested for their cy-totoxicity towards human prostate cancer cells LNCaP-C4-2B, murine melanoma cells B 16 and human breast cancer cells MDA-MB 231. (22*E*)-5α,8a-Epidioxyergosta-6,22-dien-3β-*ol* showed moderate and weak cytotoxic activity against the LNCaP-C4-2B and B 16 cell line cells (IC_50_ value of 35.53 and 78.77 μM, respectively) [128].

1,3,6-Trihydroxy-7-(dihydroxypropyl)-anthraquinone and 6-(3′-hydroxybutan-2′-yl)-3,5-dimethyltetrahydro-2*H*-pyran-2-one (**257** and **258**, Fig. 13) were isolated together with 1,3-dihydroxy-6-hydroxymethyl-7-methoxyanthraquinone, 1,3-dihydroxy-6-methyl-7-methoxy anthraquinone and biphenyl-2,2′-diyldiacetate from marine-derived fungus *Thermomyces lanuginosus* KMM 4681. The effect of all the compounds on viability and colony formation of 22Rv1 (human drug-resistant prostate cancer) was estimated. 1,3-Dihydroxy-6-methyl-7-methoxyanthraquinone exhibited cytotoxicity towards cancer cells causing, after thetreatment with 100 μM for 48 h, a reduced cell viability of 35%, while in human prostate non-cancer PNT-2 was less active (65% viability, 100 μM, 48 h). The same metabolite inhibited by 70% colony formation of prostate cancer 22Rv1 cells at the non-cytotoxic concentration of 50 μM. 22Rv1 cells are known to be drug resistant induced by the expression of AR-V7, which facilitates an autoactivation of the androgen receptor signaling mediating the resistance to enzalutamide, an androgen receptor targeting drugs. Therefore, the compounds revealing activity in AR-V7-positive prostate cancer cells are potential templates for the development of new therapeutic approaches for patient with advanced disease stages [129].

Fusarine and fusarinate (**259** and **260**, Fig. 13), two prenylated glycine derivatives, were isolated from the marine-derived fungus *Fusarium* sp. TW56-10, which was collected from Kueishantao, Taiwan. Trichodermiol A, 8-*O*-methylfusarubin, 3-*iso*-butylpyrrolopiperazine-1,4-dione, 3-phenyllactic acid methyl ester, (2*S*)-(4-hydroxyphenyl)lactic acid *N*-(2-(1*H*-indole-3-yl)ethylacetamide, indole-3-methylethanoate, (1*H*-indol-3-yl)acetic acid ethyl ester and (9*Z*,12*Z*)-*N*-(2-hydroxyethyl)-9,12-octadecadienamide were also obtained from the same fungus. Fusarine (**259**) and all the other compounds, except fusarinated (**260**) due to its low amount, were tested for their cytotoxicity towards A549 cell lines and 8-*O*-methylfusarubin showed totoxicity (IC_50_ value of 11.45 μM) [130].

**Fig. 14:**
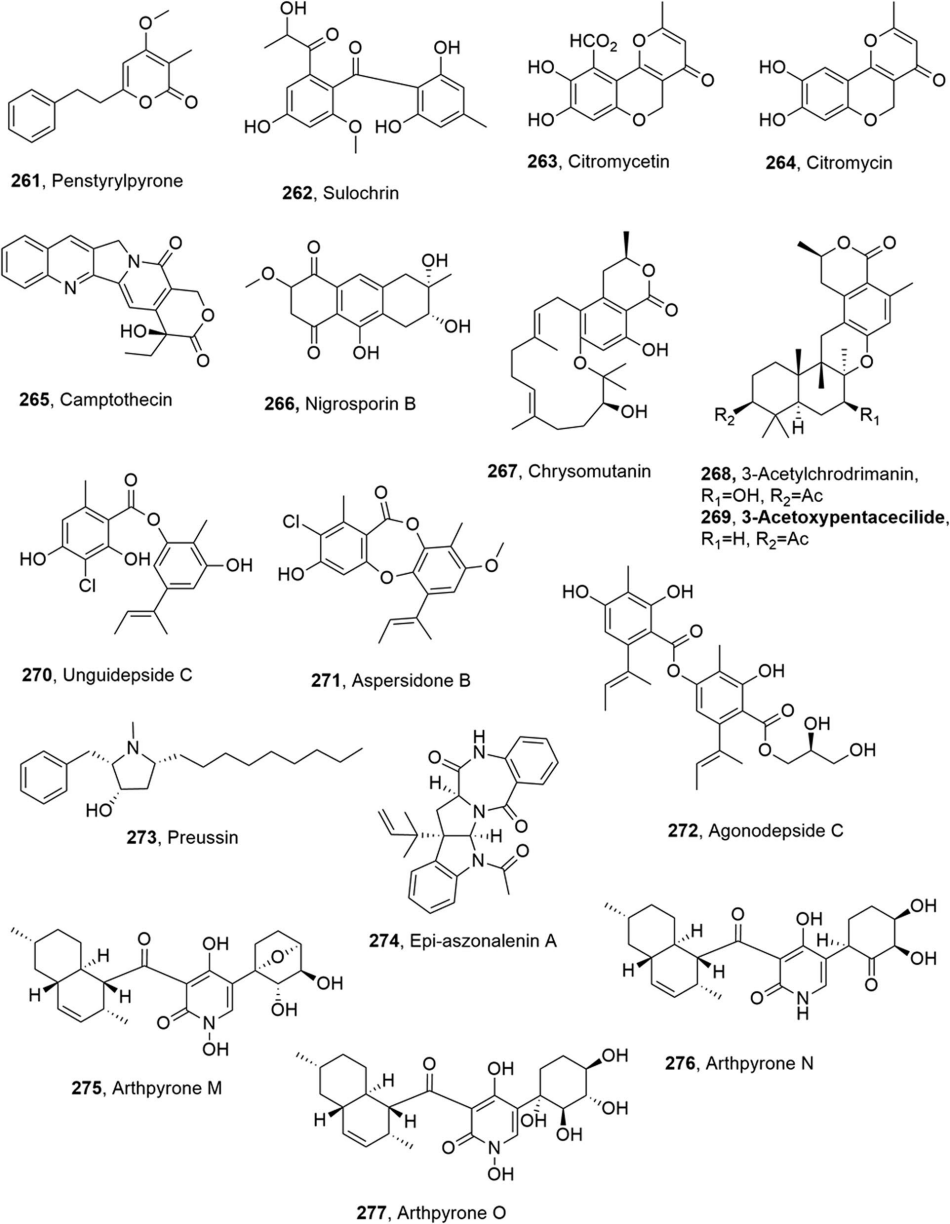
Metabolites isolated from marine fungi

Penstyrylpyrone, sulochrin, citromycetin, and citromycin (**261**–**264**, Fig. 14) were isolated from *Sporothrix* sp. SF-7266, a marine-derived fungus found in the Ross Sea around Antarctica. The anti-inflammatory, antimicrobial, and antibiotic properties of compound **261**–**264** was in deep investigated [128]. Successively, theanticancer activity of citromycin was assayed towards ovarian cancer cells. Compound **264** inhibited: (i) the migration and invasion of human ovarian cancer SKOV3 and A2780 cells, but it was not associated to cytotoxicity; (ii) the expression of epithelial–mesenchymal transition (EMT) markers and the activation of matrix metalloproteinase (MMP)-2 and MMP9; (iii) extracellular signal-regulated kinase (ERK)-1/2 signaling. Furthermore, the anti-invasive activity of citromycin was negated by the ectopic expression of ERK. These results highighted that citromycin inhibits the migration and invasion of human ovarian cancer cells by downregulation of the expression EMT markers and MMP-2/9 levels via inhibition of the ERK1/2 pathway [132].

Camptothecin (**265**, Fig. 14) was produced by the sponge-.derirerived fungus *Penicillium chrysogenum* EFBL # OL597937.1 Camptothecin displayed a strong anti-proliferative activity against HEP-2 and HCT-116 (IC_50_ values 0.33–0.35 µM). The optimization of camptothecin production via fermentation of *Penicillium chrysogenum* was also obtained [133].

Nigrosporin B (**266**, Fig. 14), an anthraquinone derivative, was isolated from the marine fungus *Nigrospora oryzae*. Nigrosporin B, in a dose-dependent manner, exhibited strong inhibition of proliferation of multiple tumor cells, and particularly against human cervical cancer Ca Ski cells (IC50 of 1.24 µM) and induced an apoptosis effect on the same cell lines. Compound 2**66** caused autophagy together with the increase of autophagic vacuoles and the acceleration of autophagic flux. The combination of nigrosporins B (**266**) with the three autophagy inhibitors strongly increased its cytotoxic activity against Ca Ski cells suggesting that the autophagy induced by compound **266** might protect Ca Ski cells from death. Nigrosporin B inhibited the phosphorylation of PI3K, AKT, mTOR molecules and induces, in a dose-dependent manner, the increasing of the protein expression levels of PTEN and p-AMPKα. These results highlighted that nigrosporins B (**266**) caused apoptosis and protective autophagy through the suppression of the PI3K/AKT/mTOR signaling pathway [134].

Chrysomutanin, 3-acetyl chrodrimanin F 4 and 5 3-acetoxypentacecilide A (**267**–**269**, Fig. 14), were isolated together with chrodrimanin F, 3-hydroxypentacecilide, chrodrimanin E, chrodrimanin B, preaustinoid A2, and (−)-preaustinoid D from a mutant of the marine-derived fungus *Penicillium chrysogenum* S-3-25 The compounds were tested for their cytotoxicity towards K562, HL-60, A549, BGC-823 and HeLa human cancer cell lines showing weak inhibitory activity excepting compounds **267**, **268** and chrodrimanin F which showed a strong inhibition on HL-60 cells (IC_50_ values of 4.8, 8.7 and 8.1 μM, respectively). The positive control, 5-FU showed inhibition rated against the tested cell lines of 71%, 72%, 51%, 55% and 73% at 100 g/mL, respectively, but the IC_50_ values were all greater than 100 μM [135].

Unguidepside C, aspersidone B and agonodepside C, (**270**–**272**, Fig. 14) were isolated from two marine-derived fungal strains of *Aspergillus unguis*. Congeners such as decarboxyunguidepide A, 2-chlorounguinol, unguinol, 3,10-dichlorounguinol, nidulin, norniduli, aspergillusidone B, aspersidone, agonodepside B, agonodepside A, guisinol, folipastatin, emeguisin A and aspergillusphenol A were also obtained from the same fungus. The compounds, except agonodepside C and aspergillusphenol A, were tested for their cytotoxic activity towards PC-3 (prostate), NCI-H23 (lung), HCT-15 (colon), NUGC-3 (stomach), ACHN (renal), and MDA-MB-231 (breast) cancer cel lines and exhibited, except aspergillusidone B, toxicity against all the tested cell lines (IC_50_ values ranging from 2.5 to 46.9 µM). The presence of a free carboxylic group determine a strong reduction of the activity, while that of the hydroxy group at C-4 is essential for cytotoxicity. The compounds **270**–**272** were also tested for their antimicrobial activity against three Gram-negative bacteria *K. pneumonia* (KCTC 2690), *Salmonella typhimurium* (KCTC 2515), and *E. coli* (KCTC 2441) and three Gram-positive bacteria *S. aureus* (KCTC 1927), *Micrococcus luteus* (KCTC 1915), and *B. subtilis* (KCTC 1021) and exhibited activity on all the tested bacteria (MIC values ranging from 5.3 to 22.1 µM) while did not inhibit the growth of Gram-negative bacteria at the concentration of 128.0 µg/mL [136].

Preussin (**273**, Fig. 14), a hydroxypyrrolidine alkaloid, was isolated from the sponge-fungus *Aspergillus candidus* KUFA 0062, collected from the Similan Islands National Park’s coral reef, Thailand. Compound **273** showed cytotoxicity towards various human breast cancer cell lines and, in particular, decreased cell viability with variable IC_50_ values, ranging from 12.3 to 74.1 µM, below 50% at 50 or 100 µM [137, 138].

The anticancer activity compound **27**3 is noteworthy against triple-negative breast cancer (TNBC), which are an aggressive subtype of breast cancer (BC) with a typically poorer prognosis than other subtypes of BC limiting therapeutic options. Preussin (**273**), in a dose-dependent manner decreased cell viability, both in 2D and 3D cell cultures, impair cell proliferation, and caused cell death, excluding the hypothesis of genotoxic properties. Preussin (**273**) also strongly inhibited the migration of MDA-MB-231 cells [139].

Epi-aszonalenin A (EAA, **274**, Fig. 14), an alkaloid, was isolated from the marine coral fungus *Aspergillus terreus* C23-3. Compound **274** through its antiangiogenic activity activated its mechanism of action against tumor metastasis and invasion. cCompound **274** interfered well with phorbol 12-myristate 13-acetate (PMA)-induced migration and invasion of HT1080 cells, decreased matrix metalloproteinase (MMPs) and vascular endothelial growth factor (VEGF) activity. It also inhibited the expression of *N*-cadherin and hypoxia-inducible factor-1α (HIF-1α) by regulating the phosphorylation of downstream mitogen activated protein kinase (MAPK), PI3K/AKT and NF-κB pathways. Furthermore, molecular docking study demonstrated that the mimic coupling between the EAA and MMP-2/-9 molecules formed a stable interaction [140].

Arthpyrones M–O (**275**–**277**, Fig. 14) were isolated together with two known pyridone derivatives, arthpyrones C and G from the sponge-derived fungus *Arthrinium arundinis *.Compounds **275**–**277** and arthpyrones C and G were tested for their cytotoxic ctivity towards five cancer cell lines and all exhibited cytotoxicity against some or all the cancer cell lines (IC_50_ values ranging from 0.26 to 6.43 μM). Furthermore, arthpyrone O (**277**) showed stong efficacy against the proliferative activity of SCLC cell lines and induced apoptosis in vitro, but also strongly inhibited the growth of xenograft tumor based on SCLC cells in vivo. These results suggested that 4-hydroxy-2-pyridone alkaloids might considered interesting in drug discovery [141].

## Conclusions

Mainly the review reports the metabolites with anticancer activity isolated in the last decade from terrestrial, including phytotpathogenic and endophytic species, and marine fungi with potential application in medicine. The well know great imagination in producing secondary metabolites showed in the past by fungi among the other microrganisms was also confirmed in the last ten years and alkaloids, terpenes, polyketides, aromatic compounds, peptides etc. with original carbon skeleton will be described. Frequently other interesting biological activities, the mode of action of anticancer metabolites and in some cases the results of SAR study and biosynthetic pathway were also reported. The metabolites isolated from terrestrial, endophytic and marine fungi belonging to different classes of naturally occurring compounds as reported in the past and last decade decribed in this review as well as probably will be in the future. Considering that only a low percentage of fungi of different origin are up to day studied to isolate and characterize their secondary metabolites the research in this field is open to significant prospectives. It represents an important tool to isolated new compounds with different biological activities for different applications in different fields as agriculture, medicine, cosmetic etc. The results of these researches could contribute to overcome the emercengy due to development of resistances arisen against pesticides, antibiotic and antitumor compounds up to day used and also could contribute to reduce the enviromental pollution due essentially to the massive use of synthetic and not biodegradable substances. The control of climate change is one of the most important problem to be solved also adopting ecofriendly methods for production in large scale of biopesticides and drugs and their distribution using the so called ‘intelligent packaging’.

## Data Availability

All the data and materials provided in the manuscript are obtained from included references and available upon request.
